# Antimicrobial resistance among agents of hospital-acquired lower respiratory tract infection in the UK and Ireland: trends from 2008/2009 to 2018/2019

**DOI:** 10.1093/jac/dkaf251

**Published:** 2025-10-27

**Authors:** Rosy Reynolds, Ian Morrissey, Shazad Mushtaq, Carolyne Horner, Rachael Adkin, Aiysha Chaudhry, Michael Allen, Christopher Longshaw, Benjamin J Parcell, David M Livermore

**Affiliations:** Population Health Sciences, University of Bristol, Bristol BS8 2PS, UK; British Society for Antimicrobial Chemotherapy, 53 Regent Place, Birmingham B1 3NJ, UK; Antimicrobial Focus Ltd., T25 Allen House, The Maltings, Station Road, Sawbridgeworth CM21 9JX, UK; Antimicrobial Resistance and Healthcare Associated Infections Reference Unit, UK Health Security Agency, Colindale, London NW9 5EQ, UK; British Society for Antimicrobial Chemotherapy, 53 Regent Place, Birmingham B1 3NJ, UK; Antimicrobial Resistance and Healthcare Associated Infections Reference Unit, UK Health Security Agency, Colindale, London NW9 5EQ, UK; Antimicrobial Resistance and Healthcare Associated Infections Reference Unit, UK Health Security Agency, Colindale, London NW9 5EQ, UK; British Society for Antimicrobial Chemotherapy, 53 Regent Place, Birmingham B1 3NJ, UK; Medical Affairs, MSD (UK) Limited, 120 Moorgate, London EC2M 6UR, UK; British Society for Antimicrobial Chemotherapy, 53 Regent Place, Birmingham B1 3NJ, UK; Scientific Affairs, Shionogi B.V., Fifty Paddington, 50 Eastbourne Terrace, Paddington W2 6LG, UK; Division of Population Health and Genomics, School of Medicine, University of Dundee, Ninewells Hospital and Medical School, Dundee DD1 9SY, UK; Department of Medical Microbiology, Ninewells Hospital and Medical School, Dundee DD1 9SY, UK; Antimicrobial Resistance and Healthcare Associated Infections Reference Unit, UK Health Security Agency, Colindale, London NW9 5EQ, UK; Norwich Medical School, University of East Anglia, Norwich NR4 7TJ, UK

## Abstract

**Objectives:**

To survey trends in antimicrobial resistance among the pathogens of hospital-acquired lower respiratory tract infection (HA-LRTI), which causes significant mortality and morbidity, particularly among ventilated patients.

**Methods:**

The BSAC Surveillance collected quotas of major HA-LRTI pathogens from sentinel sites from 2008/09 (October to September) to 2018/19. MIC testing was by BSAC agar dilution. Resistance mechanisms were inferred from synergy tests, interpretive reading and PCR.

**Results:**

Target numbers of *Staphylococcus aureus, Pseudomonas aeruginosa* and Enterobacterales—dominated by *Escherichia coli* and *Klebsiella* spp.—were reliably collected. *Acinetobacter* spp. collections were small, reflecting low incidence. Resistance rates fell or fluctuated, with no major rises. Notable declines included: (i) a fall in the proportion of MRSA among *S. aureus* from *c.* 40% to 10%; (ii) a halving, since 2012/13, in ‘triple-resistance’ to carbapenems, aminoglycosides and fluoroquinolones among *Acinetobacter baumannii sensu stricto*, from *c.* 24% to 9%; (iii) reductions in AmpC-associated cephalosporin resistance among *Enterobacter cloacae* and *Serratia* isolates, and (iv) falls in fluoroquinolone resistance among Enterobacterales, except *Klebsiella pneumoniae*. Resistance rates in *P. aeruginosa* remained low, though higher than in bacteraemia. Cephalosporin resistance in *E. coli* and *K. pneumoniae* was largely ESBL associated and, unlike AmpC-associated resistance in *Enterobacter* and *Serratia* spp., did not decline notably. Except for OXA-23 in *A. baumannii*, carbapenemases remained extremely rare. Antistaphylococcal oxazolidinones, tigecycline, ceftolozane/tazobactam, ceftazidime/avibactam and ceftobiprole retained uneroded activity.

**Conclusions:**

From 2008/09 to 2018/19, there were no major rises in resistance among the principal agents of HA-LRTI; for several important organisms/resistance combinations there were notable declines.

## Introduction

Hospital-acquired lower respiratory tract infections (HA-LRTI)—defined as pneumonia arising >48 h after hospitalization—are among the most frequent reasons for prescribing antibiotics to in-patients. Globally, their incidence is *c.* 5–10 cases per 1000 admissions.^1^ Ventilator-associated pneumonia (VAP), defined as pneumonia arising ≥48 h after intubation, arises in 2–16 cases per 1000 ventilator days among ventilated patients, where intubation facilitates bacterial colonization, giving infection rates of 10%–25%.^2^ Total mortality for VAP is high, though attributable mortality is lower, with a recent estimate of 10%.^3^ Survivors have longer hospital stays, increasing costs.^4^

*Staphylococcus aureus, Pseudomonas aeruginosa* and Enterobacterales each cause *c.* 20%–30% of HA-LRTI^5^; *Acinetobacter* and *Haemophilus influenzae* are prominent in the remaining fraction, with *Acinetobacter calcoaceticus/baumannii* (ACB) more frequent in humid countries and seasons.^6^ Among Enterobacterales, *Klebsiella pneumoniae* is prominent (and notable for resistance), along with *Enterobacter* spp., *Escherichia coli*, Proteeae and *Serratia*.^5^

Early appropriate antibiotic therapy is associated with better patient outcomes.^7,8^ However, microbiological investigation takes 48–72 h and recovers pathogens from only *c.* 50% of clinically-diagnosed cases.^9,10^ Consequently, much treatment is empirical, predicated upon local epidemiology and resistance rates.

The BSAC included HA-LRTI isolates in its surveillance from 2008/09 until 2018/19, seeking fixed quotas of *S. aureus, P. aeruginosa,* Enterobacterales and *Acinetobacter* spp. per site. This paper describes the results, showing time-trend data and outlining the activity of the newer agents tested.

## Materials and methods

Methods for the BSAC surveillances are described elsewhere in this Supplement.^11^ HA-LRTI isolates were collected in 12-month periods (October–September) from 2008/09 until 2018/19; 33–39 laboratories contributed each year from 2010/11 to 2014/15, and 21–24 in other seasons (see Tables S1–S3, available as Supplementary data at *JAC* Online, for further details). Annual quotas targeted 250–280 isolates each of *S. aureus*, *Pseudomonas* and *Acinetobacter* per centre, plus 1000–1120 Enterobacterales. Bacterial identification initially followed classical methods but increasingly moved to MALDI-TOF. MICs were determined by BSAC agar dilution and interpreted against 2022 EUCAST criteria. Selected genes (*mecA, mupA*, *bla*_CTX_ plasmid-*ampC* and those encoding carbapenemases) were sought by PCR. The antibiotics tested included core agents, tested in all seasons under the aegis of the BSAC, as well as those included for variable periods contingent on sponsorship by funders.^11^ Tables S4–S12 detail susceptibility tests by organism, antimicrobial and years included. Tables S13–S16 and Figures S1–S2 cover patient characteristics, noting any missing or non-compliant data.

So far as possible, current taxonomy is followed. Thus, isolates originally collected as *Enterobacter aerogenes* have been re-designated as *Klebsiella aerogenes*, following reclassification.^12^ We could not re-categorize isolates that, before the switch to MALDI-ToF identification, were identified only to genus level, nor those belonging to groups, e.g. the ACB complex, that were subsequently split into multiple nomenspecies.^13^

### Analysis

Analysis was descriptive and largely graphical, using Stata 18.0 (StataCorp LLC: College Station, TX, USA) and Bischoff's colour vision-sensitive ‘plotplainblind’ graph scheme.^14^ Missing data were excluded in the calculation of percentages.

## Results

### Isolate collection

**Figure 1.**
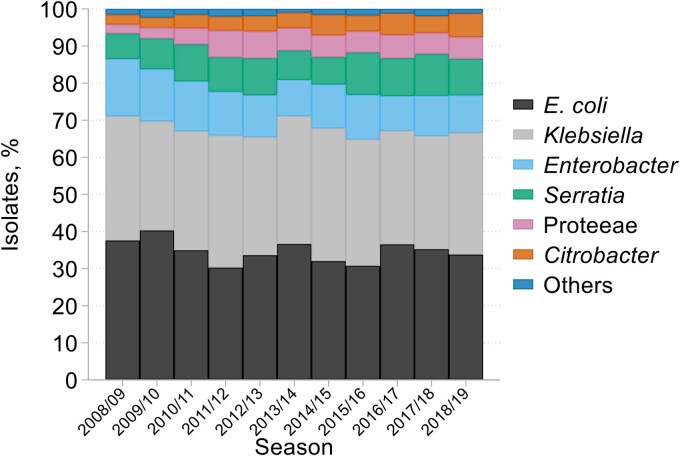
Microbial identities over time among 8183 isolates of Enterobacterales from HA-LRTI.

The collection comprised 2334 *S. aureus* (1852 MSSA, 482 MRSA, defined by presence of *mecA*), 2355 *Pseudomonas* spp., 636 *Acinetobacter* spp. and 8183 Enterobacterales (Tables S2 and S3). Enterobacterales were collected as a single group; consequently their relative proportions reflect the aetiology of HA-LRTI. They comprised 2834 *E. coli*, 2689 *Klebsiella* spp., 968 *E. cloacae* complex, 759 *Serratia* spp., 445 Proteeae (Morganellaceae), 355 *Citrobacter* and 133 isolates of other genera. *E. coli* and *Klebsiella* dominated throughout, each accounting for around a third of Enterobacterales isolates (Figure 1 and Table S3B); *Enterobacter* accounted for over half of the remainder at first, but the proportions of *Serratia* and Proteeae rose over the first few years. After 2015/16, proportions stabilized at: *c. E. coli* 34%, *Klebsiella* 32%, *Enterobacter* 11%, *Serratia* 11%, Proteeae 6%, *Citrobacter* and others (principally *Raoultella* and *Hafnia*) 7%. Adoption of MALDI-TOF-based identification did not cause any major expansion in the proportion of isolates assigned to minor genera.

In keeping with international studies,^15,16^ most source patients were male, with their proportion ranging from 59% for *Pseudomonas* to 71% for *Klebsiella* and 77% for *Citrobacter* (Table S13). The peak patient age groups were 60–69 and 70–79 years; the proportion aged ≥80 was 14%–20% except for *Acinetobacter* (6%). Infants under 1 year of age formed a distinct group, largest for *Acinetobacter* (ACB complex in 74/85 cases) and *Enterobacter*, both at 12%–13% (Table S14 and Figure S1). Proportions of isolates from ICU patients ranged from 34% for *Pseudomonas* and 39% for *S. aureus* to 48% for *Citrobacter* and 52% for *Acinetobacter*, albeit with some year-to-year change (Table S15 and Figure S2). Sputum was the source of between 60% (*Acinetobacter*) and 78% (*Pseudomonas* and Proteeae) of isolates; 11%–24% were from tracheal/endotracheal aspirates/secretions, 8%–17% from bronchoalveolar lavage and 1%–3% other sample types (Table S16).

### Staphylococcus aureus

**Figure 2.**
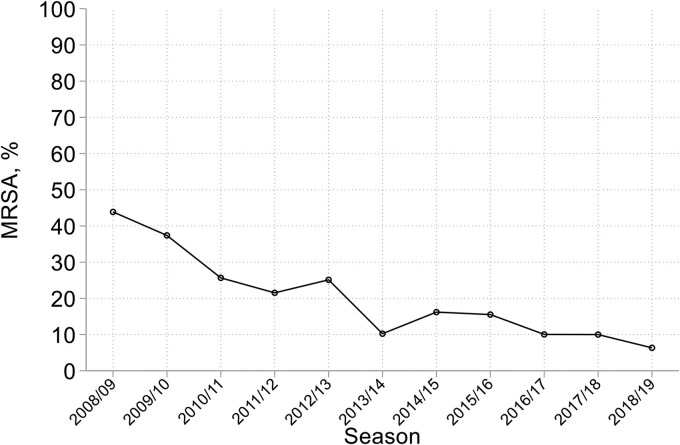
Trend in MRSA prevalence among *S. aureus* from HA-LRTI over time.

The *S. aureus* collection comprised 482 MRSA, defined by *mecA* presence, and 1852 MSSA. The MRSA proportion fell rapidly from 44% in 2008/09, then fluctuated between 16% and 6%, with a downward drift, from 2013/14 to 2018/19 (Figure 2, also Table S3A). Phenotypic resistance to oxacillin matched *mecA* status for all but 22 isolates. Four *mecA*-negative isolates with oxacillin MICs of 4 mg/L were likely BORSA (borderline oxacillin-resistant *S. aureus*) though we did not test and therefore cannot exclude the presence of *mecC*. Eighteen *mecA*-positive with oxacillin MICs of 0.12–1 mg/L (phenotypically susceptible) were inferred to have disrupted or poorly-expressed *mecA*.

**Figure 3.**
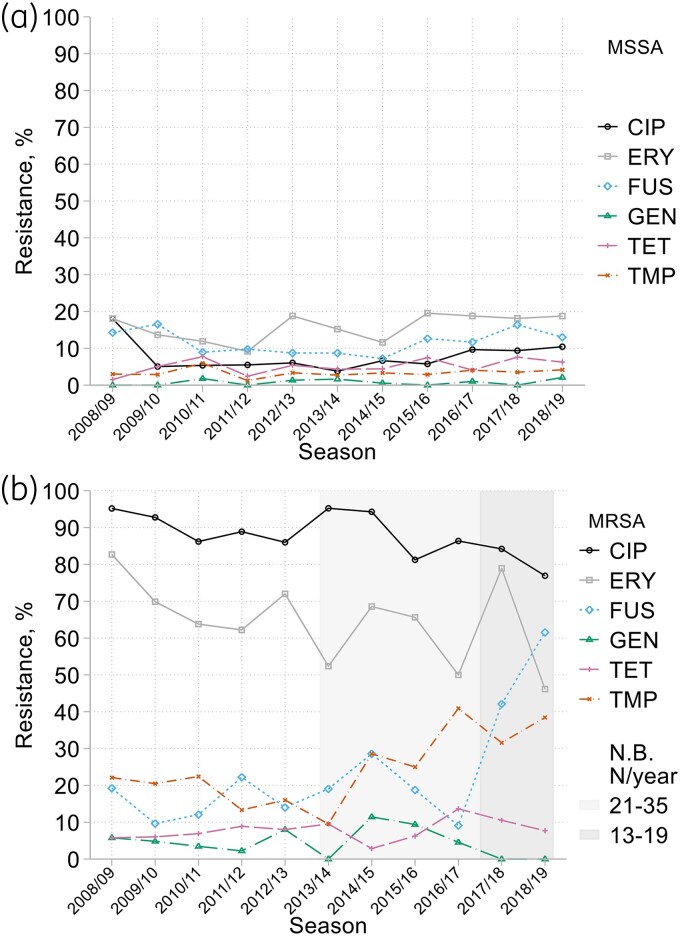
Resistance trends among (a) MSSA and (b) MRSA from HA-LRTI over time. CIP, ciprofloxacin; ERY, erythromycin; FUS, fusidic acid; GEN, gentamicin; TET, tetracycline; TMP, trimethoprim. Grey shading warns of very few MRSA isolates collected in 2013/14–2018/19 (13–35 per season).

There was little clear resistance trend among MSSA, based on 133–197 isolates/year (Figure 3a). Evidence for MRSA was limited by the low and falling numbers (104 in 2008/09; 13 in 2018/19), but results echoed those for the larger bacteraemia collections,^17^ where resistance rates fell for ciprofloxacin and erythromycin but rose for tetracycline, trimethoprim and, especially, fusidic acid (Figure 3b).

Resistances to gentamicin, rifampicin and minocycline, also high-level *mupA*-associated mupirocin resistance (MIC >64 mg/L, with *mupA* detected by PCR), were seen in <1% of MSSA but in 2% to 5% of MRSA, averaged over all years combined. Resistance was more prevalent for tetracycline (MSSA 5% versus MRSA 7%) and fusidic acid (12% versus 19%) and considerably more prevalent for ciprofloxacin (8% versus 90%), erythromycin (16% versus 69%) and trimethoprim (3% versus 22%, based on MIC >4 mg/L) (see Figure 3 and Table S4). Total clindamycin resistance (tested 2011/12 to 2018/19) tracked as slightly less prevalent than erythromycin resistance, averaging 13% for MSSA and 58% for MRSA, with 88% being erythromycin-inducible. Low-level mupirocin resistance, signified by MICs of 2–64 mg/L (mostly 8–16 mg/L) without *mupA*, was noted in 9% of MRSA and 0.1% of MSSA (see MIC distributions in Appendix to Supplementary Data). Penicillin resistance was seen in 83% of MSSA; anomalously, eight (2%) MRSA isolates appeared penicillin susceptible, though four of these were highly oxacillin resistant (MICs ≥128 mg/L).

None of the MSSA or MRSA tested was resistant to ceftobiprole, linezolid or vancomycin, fewer than 1% were resistant to tedizolid, teicoplanin or tigecycline, and fewer than 2% (a single MRSA with MIC, 2 mg/L) to ceftaroline. Modal MICs for ceftobiprole and ceftaroline were higher for MRSA (1 and 0.5 mg/L, respectively) than MSSA (0.5 and 0.25 mg/L) (see Table S4); MIC distributions for the other near-universally active agents were narrow and similar for MRSA and MSSA, with modes and (ranges): linezolid 2 (≤0.25 to 4) mg/L; tedizolid 0.25/0.5 (0.12–1) mg/L; tigecycline 0.12 (0.06–1) mg/L; teicoplanin 1 (0.12–8) mg/L; vancomycin 1 (≤0.5 to 2) mg/L (see Table S4 and MIC distributions in the Appendix to Supplementary Data).

### Pseudomonas aeruginosa

Over 99% (2335/2355) of the *Pseudomonas* isolates collected were *P. aeruginosa*. The remainder comprised 19 isolates of seven named species, mostly *P. fluorescens* (7) and *P. putida* (5), also one isolate identified only to genus level (Table S3A).

**Figure 4.**
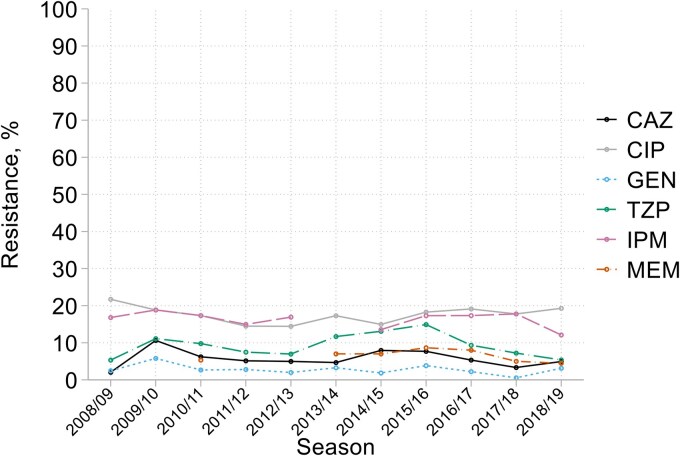
Resistance trends among *P. aeruginosa* from HA-LRTI. CAZ, ceftazidime; CIP, ciprofloxacin; GEN, gentamicin; TZP, piperacillin/tazobactam; IPM, imipenem; MEM, meropenem.

No sustained resistance trend for *P. aeruginosa* was seen, based on 179–238 (mean 212) isolates/year (Figure 4). For agents tested in all 11 years, resistance averaged 18% for ciprofloxacin, 9% for piperacillin/tazobactam, 7% for ceftazidime and 3% for gentamicin. These rates, though low, are somewhat higher than for bacteraemia.^18,19^ Addition of avibactam, tested in three years, overcame ceftazidime resistance in 22/29 cases; for 15 the ceftazidime MICs were reduced by 8- to 32-fold. Resistance to imipenem (average 16%, over 10 years) was more frequent than to meropenem (7%, seven years); addition of relebactam, tested in five years, overcame 84% (137/163) of imipenem resistance, despite a lower resistance breakpoint for the combination (2 + 4 mg/L versus 4 mg/L for imipenem alone).^20^ This wider anti-*Pseudomonas* activity of imipenem/relebactam than imipenem alone is more fully explored elsewhere.^21^ There was little resistance to amikacin (1%, based upon four years data) or tobramycin (3%, six years). Colistin and ceftolozane/tazobactam, tested over nine years, both had <1% resistance and had, respectively, 98% and 95% of MICs clustered within ±1 dilution of their modes at 1 mg/L (colistin) and 0.5 mg/L (ceftolozane/tazobactam). Ceftobiprole MICs ranged more widely, from 0.25 to ≥128 mg/L, with 85% of values within ±1 dilution of the 2 mg/L mode (see MIC distributions in Appendix to Supplementary Data).

Resistance mechanisms were sought systematically from 2013/14 to 2018/19, using the methods described^11^ together with interpretive reading.^22^ Upregulated efflux, OprD loss and AmpC hyperproduction were inferred, respectively, in 8%, 13% and 4% of the 1254 *P. aeruginosa* from this period—often in combination, and with no clear time trend. ESBL genes were confirmed in just two isolates, both from 2015/16: one had *bla*_PER_ and one *bla*_VEB_. Five isolates, scattered in time and location, had metallo-β-lactamase genes (four *bla*_VIM_ and one *bla*_NDM_).

### Acinetobacter *spp.*

**Figure 5.**
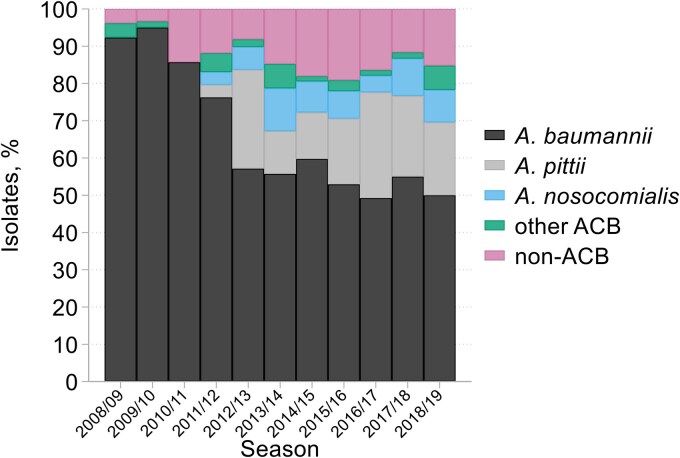
Species identifications over time among 636 isolates of *Acinetobacter* from HA-LRTI. NB, *A. baumannii* progressively becomes *A. baumannii* s.s. as other ACB species, principally *A. pittii* and *A. nosocomialis* were defined and separated. ACB, *Acinetobacter calcoaceticus*-*baumannii* complex (see text).

Despite targeting 250–280 isolates annually, only 42–72 isolates *Acinetobacter* were collected per year (mean 58/year and total 636) (Table S2). Moreover, in each year, one third to one half of the collecting laboratories failed to provide any isolates, despite mostly performing well in respect of other collection groups. Overall, 555/636 (87%) isolates were recorded as belonging to the ACB complex, comprising 416 *Acinetobacter baumannii*, 84 *Acinetobacter pittii*, 36 *Acinetobacter nosocomialis*, 17 *Acinetobacter calcoaceticus* and one each of *Acinetobacter dijkshoorniae* and *Acinetobacter seifertii*. However, four of the last five nomenspecies only came to be defined between 2011 and 2016,^13,23,24^ meaning that they are underestimated (and *A. baumannii* overestimated), in the totals. The remaining 81 isolates belonged to 13 non-ACB species, principally *A. ursingii* (20), *A. junii* (19), *A. haemolyticus* (11), *A. guillouiae* (5) *A. lwoffii* (5), *A. johnsonii* (4) and Genomic Species 16 (recently designated *Acinetobacter higginsii*) (4). The proportion of ACB complex isolates fell from ≥96% in the first 2 years to average 85% (range 82%–92%) thereafter. With MALDI-ToF identification and revised taxonomy from 2012/13 onwards, *A. baumannii sensu stricto* (*s.s.*) averaged 64% of ACB complex isolates (range 59%–73%) (Figure 5), with *A. pittii* also prominent.

**Figure 6.**
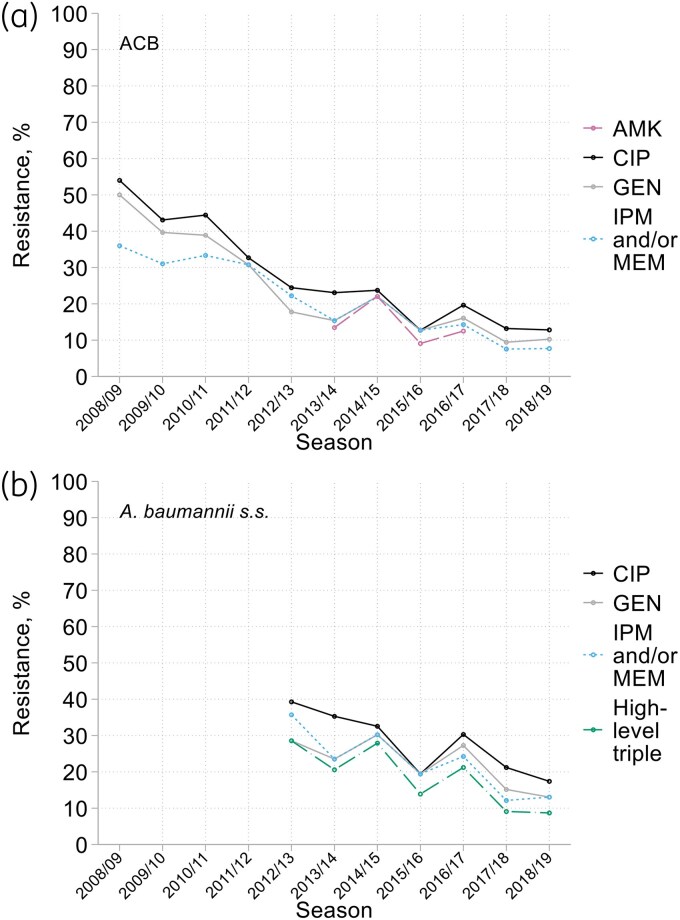
Resistance trends among (a) *Acinetobacter calcoaceticus*/*baumannii* complex and (b) *A. baumannii sensu stricto* from HA-LRTI over time. AMK, amikacin; CIP, ciprofloxacin; GEN, gentamicin; IPM, imipenem; MEM, meropenem. High-level triple resistance: resistant to CIP (MIC >1 mg/L), IPM and/or MEM (MICs >4/ >8 mg/L, respectively) and high-level resistant to GEN (MIC >64 mg/L).

Resistance was concentrated in the ACB species for 6/8 antimicrobials with EUCAST breakpoints or ECOFFs: amikacin (14% among ACB; 4% among non-ACB species), ciprofloxacin (27%; 1%), gentamicin (24%; 12%), imipenem (21%; 0%), imipenem/relebactam (13%; 0%) and meropenem (15%; 0%), with the caveat that these averages disguise falling trends (Figure 6a). Lower resistance rates for imipenem/relebactam and meropenem than imipenem reflect testing in years when imipenem resistance was less prevalent, not evasion of resistance. Colistin resistance was less frequent among ACB complex isolates (1%) than among other *Acinetobacter* species (22% overall and detected in eight different species); tobramycin resistance rates were similar, at 12%–13% among both ACB and non-ACB isolates.

Considering (i) the ACB complex only, (ii) antimicrobials tested throughout, and (iii) the period from 2012/13 onwards, when species within the complex were better discriminated, we observed that resistance was concentrated in *A. baumannii s.s.* for ciprofloxacin (28% resistant), gentamicin (23%) and carbapenems (23%), versus <2% resistance to these agents among *A. pittii* and *A. nosocomialis*. Fully 48/230 (21%) of *A. baumannii s.s.* isolates were multiresistant to carbapenems, ciprofloxacin and gentamicin, with gentamicin MICs ≥128 mg/L for 92% (44/48) of these (see MIC distributions in the Appendix to the Supplementary Data). These individual resistances approximately halved between 2012/13 and 2018/19 among *A. baumannii s.s.*, with the prevalence of high-level triple resistance falling from *c*. 24% to 9% (Figure 6b).

Resistance mechanisms were sought by PCR in isolates collected from 2013/14 to 2018/19, comprising 202 *A. baumannii s.s*, 112 other ACB species, and 60 non-ACB species. *bla*_OXA-23_ was found in 41 *A. baumannii s.s*, comprising 93% (41/43) of carbapenem-resistant *A. baumannii s.s.* isolates and 20% (41/202) of all *A. baumannii s.s.* from the period; *bla*_OXA-58_ was found in seven isolates (four *A. baumannii s.s.*, one *A. pittii* and two non-ACB isolates). *bla*_OXA-24/40_ and *bla*_OXA-143_ were not seen. Predictably, *bla*_OXA-23_ was also found in all of three *A. radioresistens* isolates, where it is endogenous.^25^

### Enterobacterales

#### Escherichia coli

**Figure 7.**
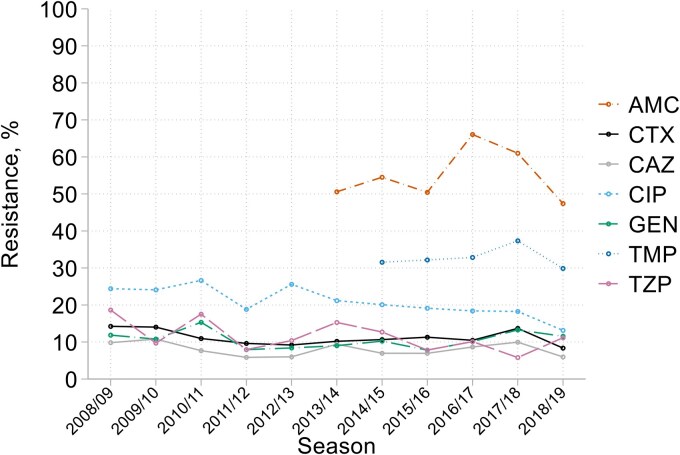
Resistance trends among *E. coli* from HA-LRTI. AMC, co-amoxiclav; CTX, cefotaxime; CAZ, ceftazidime; CIP, ciprofloxacin; GEN, gentamicin; TMP, trimethoprim; TZP, piperacillin/tazobactam.

The prevalence of resistance to amoxicillin in *E. coli* remained between 70% and 80% throughout, based on 230–295 (mean 258) isolates tested/year; co-amoxiclav resistance, tested 2012/13–18/19, ranged from 47% to 66%. Ciprofloxacin resistance was also common, though falling after 2012/13, from about 25% to consistently <20% from 2015/16 onwards, (Figure 7). There was little clear trend for cefotaxime (11% resistance overall), gentamicin (12%) and—albeit with more year-on-year fluctuation—piperacillin/tazobactam (11%). Resistance to tobramycin (8%) and trimethoprim (33%), both tested only from 2013/14 to 2018/19, showed no clear trends. Resistance rates, based upon 3–10 seasons’ testing, were <1% for eight agents described here by MIC mode and (range): amikacin 1 (0.25–32) mg/L; ceftazidime/avibactam 0.12 (≤0.008 to 0.5) mg/L; ceftolozane/tazobactam 0.12 (0.03 to ≥512) mg/L; colistin 0.5 (0.06 to ≥64) mg/L; ertapenem 0.015 (0.008–1) mg/L; imipenem 0.12 (≤0.03 to 32) mg/L; imipenem/relebactam 0.12 (≤0.03 to 1) mg/L; meropenem 0.015 (0.008–0.25) mg/L (see MIC distributions in Appendix to Supplementary Data). The higher topmost MIC for imipenem than for other carbapenems reflected a highly carbapenem-resistant carbapenemase producer being isolated in a year when only imipenem, not other carbapenems, was tested (see below). For tigecycline, resistance was <2%, with a mode MIC (range) of 0.12 (0.06 to 4 mg/L).

ESBLs were seen in 12% of all *E. coli*, tracking with cefotaxime and ceftobiprole resistances (both 11%–12%). Breakpoint-defined ceftazidime resistance (8%) averaged three points lower, consistent with the high proportion of CTX-M enzymes among ESBL-producing *E. coli* (270/348, 78%). Among these 270, 213 (79%) had group 1 CTX-M ESBLs, 52 (19%) had group 9 and five (2%) had other or multiple CTX-M types. Substantial AmpC activity, sought from 2013/14 to 2018/19 only, was inferred in 4% (58/1498) of *E. coli* isolates, compared with 181 ESBL producers (12%) in the same period.

Resistance to carbapenems was very rare. One carbapenemase-negative isolate with a CTX-M group 1 enzyme was narrowly resistant to ertapenem (MIC 1 mg/L) but susceptible to imipenem and meropenem (MICs 0.12–0.25 mg/L). PCR (2013/14 to 2018/19) identified *bla*_OXA-48-like_, in just one *E. coli*; this was susceptible to meropenem (MIC, 0.25 mg/L)—the sole carbapenem tested that year (2013/14)—but highly resistant to piperacillin/tazobactam (128 mg/L). The single isolate resistant to imipenem (MIC 32 mg/L, see above) was from 2012/13, before PCR-based testing for carbapenemase began.

### Klebsiella *spp.*

The species composition of the *Klebsiella* collections was stable over time, with *K. pneumoniae* (1656, 62%, including 52 *K. variicola*) the most frequent and *K. aerogenes* (335, 12%) the least. *K. oxytoca* (698, 26%) was more prominent in respiratory specimens than in blood, with a *K. pneumoniae: K oxytoca* ratio of 2.4:1 versus 3.5:1 for bacteraemias.^19^

**Figure 8.**
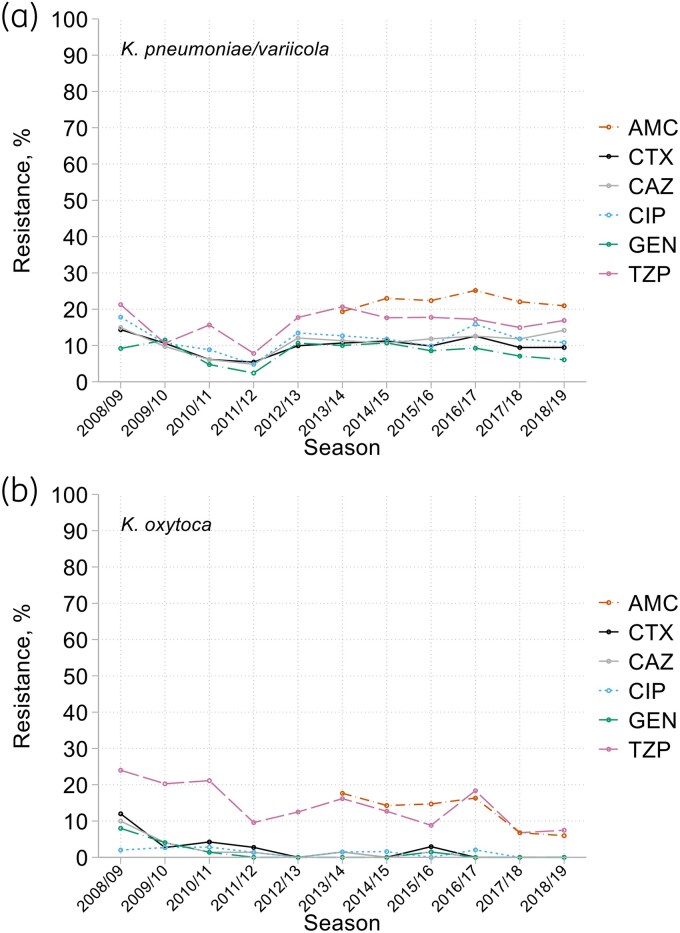
Resistance trends among (a) *K. pneumoniae*/*variicola* and (b) *K. oxytoca* from HA-LRTI. AMC, co-amoxiclav; CTX, cefotaxime; CAZ, ceftazidime; CIP, ciprofloxacin; GEN, gentamicin; TZP, piperacillin/tazobactam.

In the case of *K. pneumoniae*, and as for bloodstream isolates,^19^ resistance rates to several antibiotics dipped, then rebounded, early in the surveillance, before stabilizing from 2013/14 onwards at *c.* 10% for cefotaxime, ceftazidime and ciprofloxacin, a little lower for gentamicin, and higher for piperacillin/tazobactam (16%) and co-amoxiclav (22%) (Figure 8a). The resistance rate for ceftobiprole (15%) exceeded that for cefotaxime and ceftazidime, largely reflecting a lower breakpoint for ceftobiprole.^20^ Resistance to tobramycin (14%) was more frequent than to gentamicin; mechanisms were not investigated, but this profile is compatible with AAC(6′) variants.^22^

The smaller number of *K. oxytoca* isolates (49–74/year, mean 63) predictably produced more variable results (Figure 8b). After modest early falls, resistance remained very rare for cefotaxime, ceftazidime, ciprofloxacin and gentamicin, averaging <2% from 2013/14 onwards. Resistance to piperacillin/tazobactam fell too, though more erratically and from a higher base (>20%); from 2013/14 to 2018/19 it averaged 12%, largely matching co-amoxiclav resistance. *K. oxytoca* isolates were frequently resistant to ceftobiprole (31%), which is more affected than cefotaxime and ceftazidime by K1 β-lactamase (the chromosomal β-lactamase of the species, sometimes over-expressed) and has lower breakpoints.^20,26^ Five percent of the isolates were resistant to ertapenem; resistance to tobramycin was very rare (<1%).

Among *K. aerogenes—*based on only 16–42 isolates/year—resistance to cefotaxime (17%), ceftazidime (15%) and piperacillin/tazobactam (17%) remained prevalent from 2013/14 to 2018/19 despite apparent 6%–10% falls from 2008/09 to 2012/13. Ciprofloxacin resistance was less frequent, at 4% overall, with <1% of isolates resistant to gentamicin. Unlike other klebsiellas, *K. aerogenes* is inherently resistant to co-amoxiclav, reflecting intrinsic AmpC activity.^22^

Resistance rates to amikacin, colistin, ceftazidime/avibactam and ceftolozane/tazobactam were ≤1% for all three *Klebsiella* species except 2% for colistin in *K. pneumoniae* and 2% for ceftolozane/tazobactam in *K. pneumoniae* and *K. aerogenes*. MIC distributions for tigecycline, lacking a EUCAST breakpoint, ranged up to 8–16 mg/L, but with modes at 0.25–0.5 mg/L and only 2%–6% of isolates not inhibited at the FDA's 2 mg/L breakpoint. There were no distinct subgroups with raised tigecycline MICs (see MIC distributions in Appendix to Supplementary Data).

Patterns of resistance to β–lactams reflected different dominant resistance mechanisms in different *Klebsiella* species, each present in <2% of isolates of the other species. ESBLs predominated in *K. pneumoniae*, with an overall prevalence, based on annual estimates from 2013/14 onwards, of 12%; in *K. oxytoca,* K1 β-lactamase hyperproduction was primary, at 7% overall; in *K. aerogenes*, derepressed AmpC, recorded from 2013/14 to 2018/19, dominated (27%). Among *K. pneumoniae*, the prevalence of ESBL producers fell, then rebounded before stabilizing at 11%–15%, thus paralleling cephalosporin MIC trends. Around 62% of the ESBLs were CTX-M types, almost all of group 1; 38% were non-CTX-M types.

Carbapenemase genes were sought from 2013/14 and were detected in 10/915 *K. pneumoniae* (5 *bla*_KPC_, 3 *bla*_OXA-48-like_, and 2 *bla*_NDM_). Both isolates with *bla*_NDM_ were highly resistant to imipenem, meropenem and ertapenem, but phenotypic resistance was less consistent among those with other carbapenemase genes. Three of the five isolates with *bla*_KPC_ were resistant to both imipenem and meropenem; the other two were increased-exposure-susceptible (I) to meropenem and/or not tested for imipenem; all of the four tested with ertapenem were resistant (MICs 32 to ≥512 mg/L). All three isolates with *bla*_OXA-48-like_ appeared susceptible (S) to meropenem; one each was S, I or not tested with imipenem; both those tested with ertapenem had low-level resistance (MICs 1–2 mg/L). Imipenem/relebactam, tested in fewer years, overcame imipenem resistance in isolates with *bla*_KPC_ genes, but gave no potentiation against isolates with other carbapenemases. One *K. aerogenes* was highly resistant to imipenem (MIC, 32 mg/L) but susceptible to imipenem/relebactam (MIC 1 mg/L). This implied a Class A carbapenemase but none was found by repeated PCR, including with the extended AusDiagnostics panel.^11^ Ertapenem (tested 2014/15 to 2018/19) is more vulnerable than other carbapenems to non-carbapenemase mechanisms,^27^ leading to recognizable subpopulations with MICs around 0.06 mg/L (largely ESBL/*K. pneumoniae*), 0.03 mg/L (K1/*K. oxytoca*) and 0.5 mg/L (AmpC/*K. aerogenes*), distinct from the main modal MICs at 0.015 or 0.003 mg/L. A few isolates in these clusters counted as low-level ertapenem resistant (MICs 1–2 mg/L); as did 6/142 (4%) *K. aerogenes* and 3/765, (<1%) *K. pneumoniae*.

### Enterobacter cloacae *complex*

**Figure 9.**
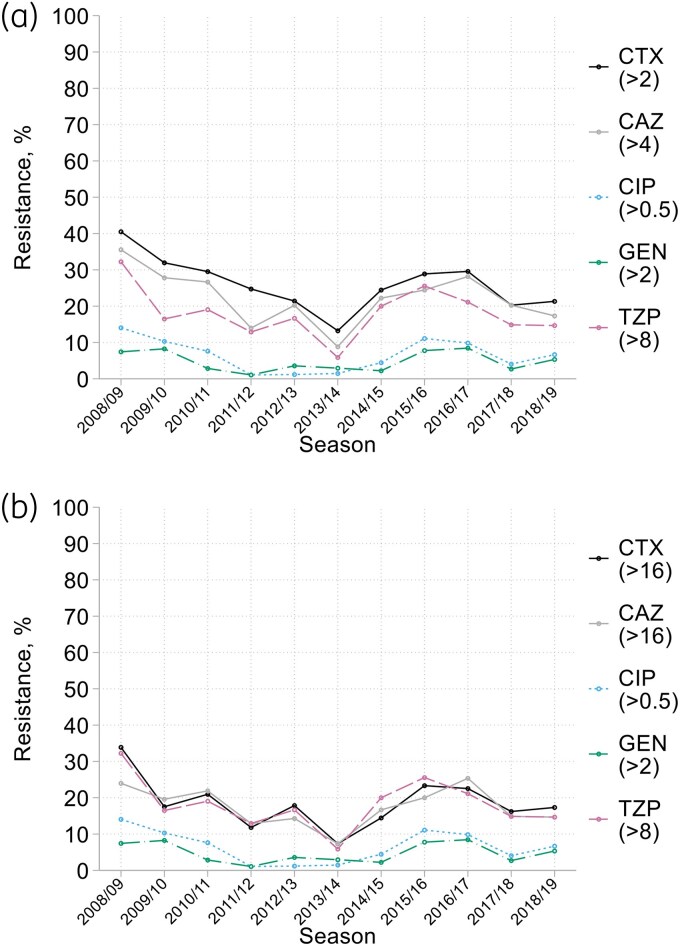
Trends in (a) resistance and (b) high-level cephalosporin resistance among *E. cloacae* complex isolates from HA-LRTI. CTX, cefotaxime; CAZ, ceftazidime; CIP, ciprofloxacin; GEN, gentamicin; TZP, piperacillin/tazobactam. The keys show the MIC conditions used to define resistance and high-level resistance (R > mg/L).

*E. cloacae* complex collections numbered 68–121 isolates/year (mean 88), totalling 968 (see Table S3B). Cefotaxime, ceftazidime and piperacillin/tazobactam all showed falling resistance trends, from *c.* 35% in 2008/09 to *c.* 10% in 2013/14, before substantially reversing over the following five years and ending around 20% (Figure 9a). This pattern was recapitulated if only isolates with cephalosporin MICs >16 mg/L were considered (Figure 9b). Similar trends, but with lower prevalence, applied for ciprofloxacin and gentamicin.

Mechanistic studies, from 2013/14 to 2018/19, recorded derepressed AmpC in 23% of isolates compared with ESBLs in 5%: these figures roughly accord with resistance rates for the same period—cefotaxime 23%, ceftazidime 21%, piperacillin/tazobactam 18%, ceftobiprole 19%, ceftolozane/tazobactam 10% and ceftazidime/avibactam (2016/17 to 2018/19 only) <1%. Across the same period, 5% of *E. cloacae* were resistant to gentamicin and 6% to tobramycin, but none to amikacin (tested 2013/14 to 2016/17 only). Tigecycline MICs ranged from ≤0.06 to 8 mg/L, with a mode 0.5 mg/L and no breakpoint. Colistin resistance occurred in 9% of isolates and was strongly associated with particular genogroups, predominantly those identified by MALDI-ToF as *E. asburiae*.^28^

The mode MIC for ertapenem (tested 2014/15 to 2018/19) was 1 mg/L for derepressed AmpC producers compared with 0.015 mg/L for non-derepressed isolates of *E. cloacae* complex, making half of the AmpC hyperproducers resistant to ertapenem in the absence of carbapenemases. Only one isolate, collected in 2017/18, was broadly resistant to carbapenems (MICs, imipenem 16 mg/L, meropenem 64 mg/L and ertapenem 64 mg/L); it had *bla*_NDM_. One further *E. cloacae*, with a likely duplicate (same centre, consecutive days in 2015/16, same patient age and sex), had *bla*_OXA-48-like_ in addition to *bla*_CTX-M_. It was resistant to ertapenem (MIC 2 mg/L) but appeared susceptible to imipenem and meropenem (MICs 1 and 0.5 mg/L).

### Serratia *spp.*

The collection of 759 *Serratia* isolates comprised 689 (91%) *S. marcescens*, 47 (6%) *S. liquefaciens*, 21 organisms of six other named species, and two not identified to species level. Proportions showed no clear time trend (see Table S3B).

**Figure 10.**
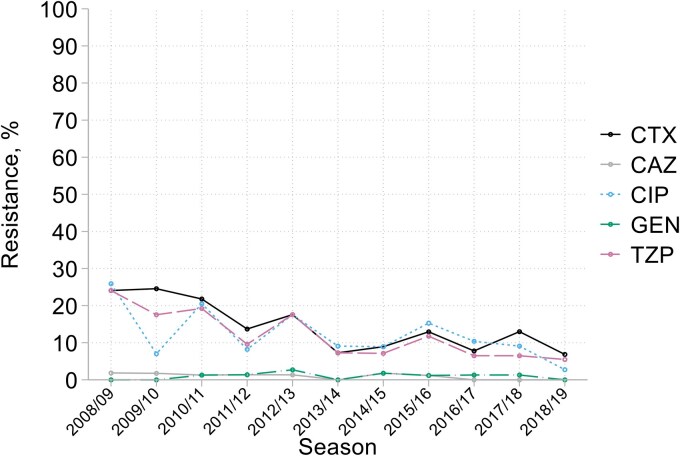
Resistance trends among *Serratia* spp. from HA-LRTI over time. CTX, cefotaxime; CAZ, ceftazidime; CIP, ciprofloxacin; GEN, gentamicin; TZP, piperacillin/tazobactam.

With the proviso of significant year-to-year noise due to small numbers (54–85 isolates/year), there were early falling trends in resistance to cefotaxime, ciprofloxacin and piperacillin/tazobactam, dropping from *c.* 20% in 2008/09 to reach plateaux of 8%–10% from 2013/14 (Figure 10). Ceftobiprole resistance was similarly prevalent in this latter period, at 11%. Resistance to gentamicin and (though tested in fewer years) amikacin, tobramycin, ceftazidime/avibactam and ceftolozane/tazobactam remained near 1%. MICs of tigecycline, lacking a breakpoint, ranged from ≤0.06 to 16 mg/L with a mode straddling 0.5–1 mg/L (see MIC distributions in Appendix to Supplementary Data).

Derepressed AmpC, sought in 2013/14 to 2018/19, was recorded in 17% of isolates, with ESBLs in *c.* 1%. Patterns of resistance among AmpC-derepressed *Serratia* differed from AmpC-derepressed *E. cloacae* complex and *K. aerogenes*, with only half being resistant to cefotaxime and ceftobiprole, none to ceftazidime and 12% to ertapenem (8/69 of those tested, 2014/15–18/19, with MICs 1–4 mg/L). One of these latter eight was resistant to imipenem (MIC, 8 mg/L) though not imipenem/relebactam (MIC 1 mg/L); all were susceptible to meropenem and were susceptible or increased-exposure-susceptible to ceftazidime. No carbapenemase genes were recorded in the 423 isolates collected from 2013/14 to 2018/19. One phenotypically carbapenem-resistant isolate, collected in 2012/13, with an imipenem MIC of 32 mg/L and high-level resistance to cefotaxime and ceftazidime (MICs ≥512 mg/L), was later found positive for *bla*_NDM_.

### *Proteeae:* Proteus, Morganella *and* Providencia

The two largest Proteeae groups were *Proteus mirabilis* (N = 354, 80%) and *Morganella morganii* (73, 16%); the remainder comprised 12 *Proteus vulgaris, 1 Proteus hauseri, 4 Providencia stuartii* and *1 Providencia rettgeri*.

The few *P. mirabilis* isolates collected (mean 40 annually, range 19–57) cautions against inference of time trends so no plots are presented. Resistance rates were substantial for amoxicillin (28% overall), ciprofloxacin (10%) and gentamicin (8%) but <2% for cefotaxime and <1% for ceftazidime and piperacillin/tazobactam. Based on three to nine years of testing, <1% resistance also was seen for ceftolozane/tazobactam, ceftobiprole and ceftazidime/avibactam. Three-quarters of gentamicin-resistant isolates were resistant also to tobramycin (tested 2013/14 onwards), but all were susceptible to amikacin (tested 2013/14 to 2016/17). Four of 320 *P. mirabilis* were resistant to imipenem at 8 mg/L (versus a mode of 2 mg/L), without evidence of carbapenemases; MICs for imipenem/relebactam (*N* = 219) were all within ±1 doubling dilution of those of imipenem. Meropenem was tested against 235 *P. mirabilis* isolates and ertapenem against 173, with no resistance seen and MIC modes of 0.06 and 0.008 mg/L, respectively. An ESBL (a non-CTX-type) was found in only one isolate; AmpC, acquired and therefore likely plasmid-determined, was inferred in 11 of 207 (5%) of *P. mirabilis* isolates collected from 2013/14 onwards, accounting for three-quarters of the 7% rate of co-amoxiclav resistance; ten had *bla*_CIT_, one *bla*_DHA._

*M. morganii* has inherent resistance to amoxicillin and co-amoxiclav but, with the caveat of the small number of isolates available, resistance rates otherwise resembled those for *P. mirabilis*, despite the potential for AmpC derepression.

### Citrobacter *spp.* and infrequent *Enterobacterales*

*Citrobacter* spp., with 355 isolates, comprised 4.3% of the Enterobacterales collected, *Raoultella* spp. (81 isolates) 1.0%, *Hafnia alvei* (37) 0.5% and 15 isolates of six further genera 0.2%.

The *Citrobacter* collection comprised 210 *Citrobacter koseri*, 122 *Citrobacter freundii*, 15 *Citrobacter braakii* plus four isolates of three other named species and three identified uncertainly as *C. koseri/amalonaticus*. The raoultellas included 51 *Raoultella ornithinolytica*, 19 *Raoultella terrigena*, 10 *Raoultella planticola* and one not identified to species level (see Table S3B).

Despite the few *Citrobacter* (19–47 isolates/year), comparison between 2008/09–12/13 and 2013/14–18/19 showed an increase in the proportion *C. koseri* (from 44% to 68%) and corresponding falls for *C. freundii* (45% to 28%) and other/uncertain species (12% to 4%).

Resistance was rare among *C. koseri,* ranging from 0% to 2% for all agents except amoxicillin (100%, inherent), piperacillin/tazobactam (11%) and co-amoxiclav (3%). Predictably, over 90% of *C. freundii* were resistant to amoxicillin and co-amoxiclav; derepressed AmpC was detected in 25% of those tested, matching 22% resistance to cefotaxime and ceftazidime. For *C. freundii*, we also recorded substantial rates of resistance for piperacillin/tazobactam and ceftobiprole (both 12%), ciprofloxacin (3%), gentamicin (5%) and tobramycin (3%) but no other tested agents (see MIC distributions in Appendix to Supplementary Data).

Information on *R. ornithinolytica* was limited by the small number of isolates, but resistance was rare except (inherently) to amoxicillin.

## Discussion

BSAC HA-LRTI surveillance began in 2008/09 whereas bacteraemia surveillance commenced seven years earlier. Consequently, and unlike the bacteraemia surveillance, the HA-LRTI Programme missed the rise in CTX-M-ESBL-producing Enterobacterales that occurred around 2002–06.^19^ It also began once MRSA was in decline in the UK,^29^ and 5–6 years after the national spread of carbapenem-resistant *A. baumannii*.^30^ What the surveillance did capture was the decline of these and other resistances.

The proportion of MRSA among *S. aureus* fell throughout, rapidly at first and then more gradually, lagging 2–3 years behind similar declines for bacteraemia.^17^ At initiation in 2008/09 the HA-LRTI surveillance registered an MRSA rate >40% whereas the corresponding bacteraemia rate was 22%–25%, having fallen below 40% in 2007.^17^ When the BSAC surveillances ended, in 2018/19 and 2019, both component Programmes were recording MRSA rates below 10%, as was UKHSA bacteraemia surveillance.^17^ It remains uncertain whether the HA-LRTI MRSA rate was even higher before 2008/09 or if the decline began later than for bacteraemia.

In the case of *P. aeruginosa*, resistance rates were stable throughout, as in bacteraemia.^19^ They tended to be higher than among bloodstream isolates^18^ (e.g. 18% versus 8%, 16% versus 6% and 6% versus 2% for ciprofloxacin, imipenem and ceftazidime, respectively in the last five surveillance seasons) probably reflecting inclusion of organisms from patients with underlying chronic respiratory colonization and repeated antibiotic exposure.^18^

For *Acinetobacter*, analysis is complicated: (i) by the greater discriminatory power of MALDI-TOF-based identification, adopted from 2011/12, replacing the API20NE strips used previously, and (ii) by the division of the ACB complex into multiple nomenspecies.^13,23,24^ It is tempting to think that a rise in non-ACB species, from <5% in 2008/09 and 2009/10 to 8%–19% thereafter might reflect the changed identification method. However, this seems not to be the case, because the shift largely occurred a year before the adoption of MALDI-TOF. What MALDI-TOF did do was to enable identification of newly-designated ACB complex nomenspecies showing that, from 2012/13 onwards, *c.* 64% of all ACB complex were *A. baumannii s.s.*, 23% *A. pittii* and 9% *A. nosocomialis*. *A. baumannii s.s.* isolates with OXA-23 carbapenemase accounted for almost all ‘triple resistance’ to carbapenems, ciprofloxacin and high-level gentamicin. Given that this profile corresponds to the OXA-23 clones 1 and 2, which accounted for most carbapenem resistant acinetobacters early in this century,^30^ it is reasonable to interpret falling multi-resistance (Figure 6) as the decline of these lineages. Reasons are uncertain but plausibly include reinforcement of hospital infection control,^31^ reduced use of fluoroquinolones,^32^ and clonal ‘exhaustion’, reflecting, e.g. increasing degeneracy and/or proliferation of bacteriophage.

Resistance among HA-LRTI Enterobacterales changed little and erratically, or trended downwards. Downward trends were seen for cephalosporin resistance in *E*. *cloacae* (albeit with a partial reversal after 2013/14) and *Serratia* spp., also for ciprofloxacin resistance among *Serratia* spp. and *E. coli*, though not *Klebsiella* spp. and *Enterobacter* spp. As in bacteraemia,^19^ it is likely these declines reflected altered antibiotic usage. Since *c.* 2005, the UK has reduced fluoroquinolone use, concerned that these agents were a major selector for *Clostridioides difficile*, thereby also mitigating selection pressure on other species.^32^ Similarly, there has been a move away from oxyimino cephalosporins, which notoriously select for AmpC-derepressed mutants of *E. cloacae*, *K. aerogenes*, *C. freundii* and *Serratia* spp.^33,34^ Reduced usage should reduce *de novo* selection, explaining the trends seen. There was much less evidence of downtrends for resistances that mostly are plasmid encoded, as with ESBL-mediated cephalosporin resistance in *E. coli* and *K. pneumoniae*. Despite considerable concern about carbapenemase producers, these were encountered infrequently, with no evidence of the accumulation seen in eastern and southern Europe^35^ and no one type dominant.

Despite this broadly encouraging picture, several high resistance rates deserve highlighting as potentially compromising empirical therapy. Resistance to co-amoxiclav was seen in around 50% of *E. coli* and 20% of *K. pneumoniae* and is inherent in *Enterobacter* and *Serratia* spp. This agent’s empirical use in HA-LRTI should be avoided. Next, there has been some stewardship-based encouragement of trimethoprim (or co-trimoxazole) in secondary care.^36^ The present data counsel caution, at least in respect of any empirical use: *c.* 33% of *E. coli* were trimethoprim resistant, even at uncomplicated urinary breakpoints, as were a rising proportion of MRSA.

Agents launched during the past quarter century have retained activity. Among MRSA, minimal resistance was seen for linezolid, tedizolid, tigecycline, ceftaroline and ceftobiprole. Retention of activity by oxazolidinones is especially notable, 25 years after linezolid's introduction and the first recognition of mutational resistance via 23S rDNA modification.^37^ Accumulation of resistance has likely been retarded by its instability unless multiple internal recombination events follow. No trend to increasing resistance was seen among Enterobacterales for ceftazidime/avibactam, ceftolozane/tazobactam or ceftobiprole, though all have some gaps in their coverage, notably metallo-carbapenemase producers for ceftazidime/avibactam and the great majority of carbapenemase producers for ceftolozane/tazobactam and ceftobiprole. Retention of good activity by these agents is in keeping with UK reference laboratory experience, with the caveat that ceftolozane/tazobactam is undermined against ESBL producers with increased impermeability.^38^ Tigecycline MIC distributions remained unaltered over the years tested, though EUCAST now only retains breakpoints for *E. coli* and *C. koseri* among Gram-negatives.^20^ Against *P. aeruginosa*, ceftolozane/tazobactam had lower MICs than ceftazidime, including—as expanded elsewhere—for many with AmpC or efflux type resistance^38^; ceftazidime/avibactam overcame around three-quarters of ceftazidime resistance, implying that much involved AmpC, even when our interpretive reading principally indicated efflux. Imipenem/relebactam overcame most carbapenem-independent imipenem resistance, which requires functional AmpC together with loss of porin OprD.^21^

A caveat to this surveillance is that pathogens typically are recovered from only *c.* 50% of patients clinically suspected of having LRTI, with considerable inter-hospital variation.^9^ Multiplex PCR, performed directly on respiratory secretions, detects organisms in more patients than culture, though their clinical significance can be debated.^9^ A further caveat concerns the COVID-19 pandemic, beginning one year after the end of the BSAC surveillance. During COVID-19's first wave, ventilated COVID-19 patients were disproportionately infected with *K. pneumoniae* and *P. aeruginosa*, increasing the relative importance of these opportunist secondary pathogens in HA-LRTI.^39,40^ It is likely that the epidemiology has now re-normalised, with SARS-CoV-2 endemic and rarely precipitating ICU admission. Nevertheless, direct confirmation is lacking, underscoring the need for future surveillance.

## Supplementary Material

dkaf251_Supplementary_Data

## Transparency declarations

S.M., R.A. and A.C. are employees of the UKHSA and work for the Antimicrobial Resistance and Healthcare Associated Infections Reference Unit (AMRHAI), which has received financial support for conference attendance, research projects or contracted evaluations from numerous sources, including Accelerate Diagnostics, Allecra Therapeutics, AstraZeneca UK Ltd, AusDiagnostics, Basilea Pharmaceutica, Becton Dickinson Diagnostics, bioMérieux, Bio-Rad Laboratories, BSAC, Cepheid, Check-Points B.V., UK Department of Health, Enigma Diagnostics, Food Standards Agency, GlaxoSmithKline Services Ltd, Helperby Therapeutics, Henry Stewart Talks, IHMA Ltd, Innovate UK, Integra holdings, Merck Sharpe & Dohme Corp., Meiji Seika Pharma Co. Ltd, Mobidiag, Momentum Biosciences Ltd, Neem Biotech, Nordic Pharma Ltd, Norgine Pharmaceuticals, Paratek Pharmaceuticals, Roche, Smith & Nephew UK Ltd, Shionogi & Co. Ltd, Trius Therapeutics, VenatoRx Pharmaceuticals and Wockhardt Ltd. M.A. is a Trustee of the BSAC Council and is employed by MSD (UK) Limited, London, UK. M.A. has shareholdings in Merck & Co., Inc., Rahway, NJ, USA. C.L. is a Trustee of the BSAC Council and is employed by Shionogi B.V. I.M. is Founder and Consultant at Antimicrobial Focus Ltd, which provides consulting services for multiple pharma and diagnostic companies. C.H. was employed by Una Health Limited and QuantuMDx Group Limited during the period of data analysis and manuscript preparation. B.J.P.: consultancy: Genpax. D.M.L.: Advisory Boards or *ad hoc* consultancy: AdjuTec, AstraZeneca, Centauri, GenPax, GSK, Lipovation, Meiji, Paion, Shionogi, Sumitovant, Thermo Fisher and Wockhardt; Paid lectures—bioMérieux, Pfizer, Shionogi and Zuellig Pharma. Relevant shareholdings or options—GenPax, GSK, Merck & Co. Inc., and Revvity amounting to <10% of portfolio value. D.M.L. also has shareholdings in Arecor, Genincode, Probiotix, Rua Life Sciences, SkinBiotherapeutics and VericiDx (all with research/products pertinent to medicines or diagnostics) through Enterprise Investment Schemes, but has no authority to trade these holdings directly. All other authors have nothing to declare.

## Supplementary data

Figures S1 to S2 and Tables S1 to S16 and Appendix are available as Supplementary data at *JAC* Online.
