# Supplementary material for: Antimicrobial resistance among agents of hospital-acquired lower respiratory tract infection in the UK and Ireland: trends from 2008/2009 to 2018/2019
Source: J Antimicrob Chemother. 2025 Oct 27;80(Suppl 4):iv49–59. doi: 10.1093/jac/dkaf251 (PMC12555202; doi:10.1093/jac/dkaf251)
Supplement: dkaf251_Supplementary_Data [file dkaf251_supplementary_data.pdf]

Antimicrobial resistance among agents of hospital-acquired lower respiratory tract infection in the UK and Ireland: trends from 2008/2009 to 2018/2019

SUPPLEMENTARY INFORMATION

Methods for the BSAC Resistance Surveillance Project are described in detail in a companion paper.[1] Breakpoints and ECOFFs (Epidemiological Cut-offs) listed in Tables S4–S11 are from EUCAST tables of breakpoints (v12.0) and related guidance at the time of analysis (https://www.eucast.org), specifically:

> European Committee on Antimicrobial Susceptibility Testing. **Breakpoint tables** for interpretation of MICs and zone diameters. Version 12.0, valid from 2022-01-01.". https://www.eucast.org; https://www.eucast.org/fileadmin/src/media/PDFs/EUCAST_files/Breakpoint_tables/v_12.0_Breakpoint_Tables.pdf. Accessed 20 January 2025
>
> European Committee on Antimicrobial Susceptibility Testing. Guidance document: **EUCAST breakpoints in brackets** 1 December, 2021. Available at: https://www.eucast.org/fileadmin/src/media/PDFs/EUCAST_files/Guidance_documents/Breakpoints_in_brackets.pdf /. Accessed 13 January 2025.
>
> European Committee on Antimicrobial Susceptibility Testing. Data from the EUCAST MIC distribution website. https://www.eucast.org/mic_and_zone_distributions_and_ecoffs; https://mic.eucast.org/; https://mic.eucast.org/search/.

Contents

BSAC hospital-acquired LRTI resistance surveillance – isolates and tests 3

Table S1. Isolate collection quotas and targets – BSAC HA-LRTI surveillance.......... 3

Table S2. Actual numbers of isolates tested, and centres contributing, by year – BSAC HA-LRTI surveillance.......... 3

Data amendments, compliance and change of central testing laboratory.......... 3

Table S3A. Isolates other than Enterobacterales – numbers tested by organism group and year 4

Table S3B. Enterobacterales – numbers tested by organism group and year. 5

Table S4. *S. aureus*: antibiotics analysed – years included, N of isolates, resistance breakpoints and mode MIC 6

Table S5. *P. aeruginosa*: antibiotics analysed – years included, N of isolates, resistance breakpoints and mode MIC 7

Table S6. *Acinetobacter* spp.: antibiotics analysed – years included, N of isolates, resistance breakpoints and mode MIC.......... 8

Table S7. *E. coli*: antibiotics analysed – years included, N of isolates, resistance breakpoints and mode MIC 9

Table S8. *Klebsiella* spp: antibiotics analysed – years included, N of isolates, resistance breakpoints and mode MIC 10

Table S9. *E. cloacae* complex: antibiotics analysed – years included, N of isolates, resistance

breakpoints and mode MIC 11
Table S10. *Serratia* spp: antibiotics analysed – years included, N of isolates, resistance breakpoints and mode MIC 12
Table S11. Proteeae: antibiotics analysed – years included, N of isolates, resistance breakpoints and mode MIC 13
Table S12. *Citrobacter* spp. and less frequent Enterobacterales: antibiotics analysed – N of years included, N of isolates, resistance breakpoints and mode MIC 15
BSAC hospital-acquired LRTI resistance surveillance – patient characteristics 16
Sex 16
Table S13. Proportion (%) of male patients by organism species 16
Age 17
Table S14. Patient age: summary measures by organism 17
Figure S1. Patient age: histograms and trends by organism group 18
Care setting: intensive/critical care speciality (ICU) 20
Table S15. Proportion (%) of isolates from ICU patients, by organism 20
Figure S2. Trends in proportion of isolates from ICU patients, by organism group 21
Specimen type 22
Table S16. Specimen types – % by organism 22
References 23
APPENDIX – MIC distributions 24
S. aureus – MSSA & MRSA 25
P. aeruginosa 28
Acinetobacter calcoaceticus-baumannii (ACB) complex 29
E. coli 30
Klebsiella spp. 31
E. cloacae complex 34
Serratia 36
P. mirabilis 37
M. morganii 38
Citrobacter spp. 40

BSAC hospital-acquired LRTI resistance surveillance – isolates and tests

Table S1. Isolate collection quotas and targets – BSAC HA-LRTI surveillance

Annual collection periods[1]	Target N of centres	Enterobacterales Quota/lab	Enterobacterales Target	All other collection groups[2] Quota/lab	All other collection groups[2] Target
2008/09–2009/10	20	50	1000	13	260
2010/11–2014/15	40	28	1120	7	280
2015/16–2018/19	25	40	1000	10	250

[1] October–September
[2] *Staphylococcus aureus*, *Pseudomonas* spp., *Acinetobacter* spp.

Collections per participating centre per season averaged 84% of the requested quota of HA-LRTI isolates for *S. aureus* and *Pseudomonas* spp., 74% for Enterobacterales but only 23% for the much-less-frequent *Acinetobacter* spp.

Table S2. Actual numbers of isolates tested, and centres contributing, by year – BSAC HA-LRTI surveillance

Collection season	N of centres[1]	N of isolates *S. aureus*	*Pseudomonas*	*Acinetobacter*	Enterobacterales
2008/09	21	237	244	52	785
2009/10	22	222	207	60	690
2010/11	38	226	225	42	784
2011/12	35	209	214	59	789
2012/13	33	199	201	49	744
2013/14	37	205	214	61	696
2014/15	39	216	214	72	762
2015/16	24	206	208	68	748
2016/17	24	219	225	67	758
2017/18	24	190	180	60	684
2018/19	24	205	223	46	743
Total	(65)	2,334	2,355	636	8,183

[1] Number of centres that contributed any isolates from HA-LRTI in that season: not all contributed isolates of all organism groups. In total, only 45 of the 65 centres in total contributed any isolates of *Acinetobacter* spp., whereas the other three collections each included isolates from 63 or 64 sites.
See tables S3A and S3B for genera, species or other subgroups (e.g. MSSA/MRSA) of these four collection groups.

Data amendments, compliance and change of central testing laboratory

Five *S. aureus* isolates originally recorded as *mecA*-negative were noted as having discrepantly high MICs for oxacillin (≥32 mg/L) during data review for this paper. They were retested in 2024. All were found *mecA*-positive on retesting and, accordingly, were reclassified as MRSA.

A very small proportion of isolates were included in analysis despite departures from protocol requirements for samples to be taken >48 hours after hospital admission and from the lower respiratory tract. These are noted where relevant – see above Table S15 and below Table S16).

Central testing was at Quotient Bioresearch Ltd (later LGC) Fordham, UK in 2008/09–2012/13 and at the Antimicrobial Resistance and Healthcare-Associated Infections Reference Unit (AMRHAI) of Public Health England, later the UK Health Security Agency (Colindale, London) 2013/14–2018/19.

Table S3A. Isolates other than Enterobacterales – numbers tested by organism group and year

Collection year	N of isolates					
	S. aureus		*Pseudomonas*		*Acinetobacter*	
	MSSA	MRSA	*P. aeruginosa*	Other species[1]	ACB complex[2]	Other species[3]
2008/09	133	104	238	6	50	2
2009/10	139	83	206	1	58	2
2010/11	168	58	223	2	36	6
2011/12	164	45	213	1	52	7
2012/13	149	50	201	0	45	4
2013/14	184	21	212	2	52	9
2014/15	181	35	211	3	59	13
2015/16	174	32	207	1	55	13
2016/17	197	22	223	2	56	11
2017/18	171	19	179	1	53	7
2018/19	192	13	222	1	39	7
Total	1,852	482	2,335	20	555	81

[1] 19 isolates belonging to seven species (7 *P. fluorescens*; 5 *P. putida*; 2 each of *P. koreensis*, *P. mendocina*; 1 each of *P. monteilii*, *P. rhodesiae*, *P. stutzeri*) and 1 identified only as *Pseudomonas* spp.

[2] Recorded as 416 *A. baumannii*, 84 *A. pittii*, 36 *A. nosocomialis*, 17 *A. calcoaceticus*; 1 *A. dijkshoorniae*, 1 *A. seifertii*; however the last four species were not defined in the earlier years and would have been counted as *A. baumannii*, inflating the total for this species.

[3] 13 different species, the most frequent being *A. ursingii* (20), *A. junii* (19) and *A. haemolyticus* (11) *A. guillouiae* (5) *A. lwoffii* (5), *A. johnsonii* (4) and genomic species 16 (4).

Table S3B. Enterobacterales – numbers tested by organism group and year.

Year		*Klebsiella*				*Serratia*[3]			Proteeae				*Citrobacter and infrequent genera*				
	E. coli	*K. pneumoniae / variicola*	*K. oxytoca*	*K. aerogenes*[1]	*E. cloacae* complex[2]	*S. marcescens*	*S. liquefaciens*	*Serratia*, other or uncertain species[3]	*Proteus mirabilis*	*Proteus*, other species[4]	*Morganella morganii*	*Providencia* spp.[5]	*Citrobacter koseri*	*Citrobacter freundii*	*Citrobacter*, other or uncertain species[6]	*Raoultella* spp.[7]	Other genera[8]
2008/09	295	174	50	39	121	47	5	2	11	1	7	0	7	9	5	7	5
2009/10	278	113	74	16	97	49	7	1	16	1	3	0	6	10	3	12	4
2010/11	274	147	71	34	105	71	3	4	28	0	6	0	14	10	5	6	6
2011/12	239	166	73	42	93	61	2	10	45	4	7	1	14	15	1	12	4
2012/13	250	141	56	40	84	74	0	0	47	1	6	0	16	14	1	7	7
2013/14	255	150	68	22	68	51	4	0	34	0	7	1	20	7	2	1	6
2014/15	244	187	63	23	90	49	6	1	37	1	7	0	34	7	1	5	7
2015/16	230	152	68	35	90	79	6	0	37	1	5	0	23	9	0	9	4
2016/17	277	151	49	32	71	72	4	1	36	2	8	2	30	12	2	6	3
2017/18	241	127	59	23	74	70	6	1	28	0	10	1	16	14	1	8	5
2018/19	251	148	67	29	75	66	4	3	35	2	7	0	30	15	2	8	1
Total	2,834	1,656	698	335	968	689	47	23	354	13	73	5	210	122	23	81	52

Numbers are shown after reclassifying isolates retrospectively where required by changes in taxonomy during the course of the surveillance.

[1] 300 of the *K. aerogenes* (2008/09–2017/18) were originally recorded as *E. aerogenes*.

[2] All the Enterobacter isolates remaining after taxonomic reclassification belonged to species within the *E. cloacae* complex: 2763 *E. cloacae*, 172 "*E. cloacae* complex", 112 *E. asburiae*, 13 *E. bugandensis*, 4 *E. kobei*.

[3] 11 *S. ureilytica*, 10 of five other named species, 2 identified only to genus level.

[4] 12 *P. vulgaris*, 1 *P. hauseri.*

[5] 4 *P. stuartii, 1 P. rettgeri.*

[6] 15 *C. braakii, 3 C. murliniae, 1 C. amalonaticus,* 4 identified uncertainly as *C. koseri/amalonaticus.*

[7] 15 *R. ornithinolytica*, 19 *R. terrigena*, 10 *R. planticola*; 1 identified only to genus level.

[8] 37 *Hafnia alvei*; 15 isolates of six other genera, of which 8 were collected as *Enterobacter* but have since been reclassified.[2]

Table S4. *S. aureus*: antibiotics analysed – years included, N of isolates, resistance breakpoints and mode MIC

Antimicrobial	Collection years included	N of years	Break-point R > mg/L	MSSA		MRSA	
				N of isolates	Mode MIC mg/L	N of isolates	Mode MIC mg/L
Ceftaroline	2016/17–2018/19	3	1	560	0.25	54	0.5
Ceftobiprole	2011/12–2017/18	8	2	1412	0.5	237	1
Ciprofloxacin	2008/09–2018/19	11	1	1852	0.5‡	482	128‡
Clindamycin[1]	2008/09–2018/19	11	0.25	1852	0.12‡	482	0.12‡
Erythromycin	2008/09–2018/19	11	2	1852	0.25‡	482	≥256‡
Fusidic acid	2008/09–2018/19	11	1	1852	0.12‡	482	0.12‡
Gentamicin[2]	2008/09–2018/19	11	2	1852	0.25	482	0.25‡
Linezolid	2008/09–2011/12; 2018/19	6	4	945	2	353	2
Minocycline	2008/09–2014/15	7	0.5	1118	0.12‡	396	0.12‡
Mupirocin[3]	2008/09–2018/19	11	1	1852	0.25	482	0.25‡
Oxacillin[4]	2008/09–2018/19	11	2	1852	0.25	482	≥256‡
Penicillin	2008/09–2014/15	7	0.12	1118	≥128‡	396	64
Rifampicin	2008/09–2018/19	11	0.06	1852	0.008‡	482	0.008‡
Tedizolid	2014/15–2018/19	5	0.5	915	0.25	121	0.5
Teicoplanin	2008/09–2018/19	11	2	1852	1	482	0.5
Tetracycline	2008/09–2018/19	11	2	1852	0.5‡	482	0.5‡
Tigecycline	2008/09–2012/13	5	0.5	753	0.12	340	0.25
Trimethoprim[5]	2008/09–2018/19	11	4	1852	0.5‡	482	0.25‡
Vancomycin	2008/09–2018/19	11	2	1852	1	482	≤0.5

‡ Most frequent MIC, but distribution was clearly bi- or multi-modal: refer to plot in Appendix.

[1] Clindamycin resistance is reported at face value using this 2022 EUCAST breakpoint (MIC >0.25 mg/L). Inducible resistance was tested (2012–2019) with 4 mg/L erythromycin plus 0.5 mg/L clindamycin, the then breakpoint, so may be slightly underestimated relative to the lower 2022 breakpoint.

[2] Epidemiological cut-off (ECOFF) 'breakpoint in brackets', to distinguish isolates with acquired resistance mechanisms likely to undermine effectiveness when used as a synergist in combination therapy.

[3] ECOFF, for descriptive purposes. Mupirocin resistance was further assessed by PCR for *mupA*.

[4] Phenotypic oxacillin resistance was used to identify possible BORSA; MRSA was defined by detection of *mecA*.

[5] Trimethoprim is of limited relevance outside UTI, but co-trimoxazole occasionally is advocated for wider NHS use. [3,4]

Table S5. *P. aeruginosa*: antibiotics analysed – years included, N of isolates, resistance breakpoints and mode MIC

Antimicrobial	Collection years included	N of years	Break-point R > mg/L	*P. aeruginosa*	
				N of isolates	Mode MIC mg/L
Amikacin[1]	2013/14–2016/17	4	16	853	2
Ceftazidime	2008/09–2018/19	11	8	2335	2
Ceftazidime/avibactam[2]	2016/17–2018/19	3	8	624	2
Ceftobiprole	2011/12–2018/19	8	-	1668	2
Ceftolozane/tazobactam[2]	2010/11–2018/19	9	4	1891	0.5
Ciprofloxacin	2008/09–2018/19	11	0.5	2335	0.12‡
Colistin[1]	2010/11–2018/19	9	4	1891	1
Gentamicin[3]	2008/09–2018/19	11	8	2335	1‡
Imipenem	2008/09–2012/13; 2014/15–2018/19	10	4	2123	1‡
Imipenem/relebactam[2]	2014/15–2018/19	5	2	1042	0.25
Meropenem	2010/11; 2013/14–2018/19	7	8	1477	0.25‡
Piperacillin/tazobactam[2]	2008/09–2018/19	11	16	2335	4‡
Tobramycin[1]	2013/14–2018/19	6	2	853	0.5‡

20 isolates of other *Pseudomonas* species are omitted.

‡ Most frequent MIC, but distribution was clearly bi- or multi-modal: refer to plot in Appendix.

[1] Epidemiological cut-off (ECOFF) 'breakpoint-in-brackets', intended to distinguish isolates with acquired resistance mechanisms likely to undermine effectiveness when used as a synergist in combination therapy.

[2] Tested with a fixed 4 mg/L concentration of inhibitor (avibactam, relebactam or tazobactam).

[3] Gentamicin has no breakpoint-in-brackets in EUCAST's tables. We used the ECOFF as an unofficial indicator of likely acquired resistance.

Table S6. *Acinetobacter* spp.: antibiotics analysed – years included, N of isolates, resistance breakpoints and mode MIC

Antimicrobial	Collection years included	N of years	Break-point R > mg/L	*ACB complex* N of isolates	Mode MIC mg/L
Amikacin[1]	2013/14–2016/17	4	8	222	0.5‡
Ceftazidime	2008/09–2018/19	11	x	555	4‡
Ceftazidime/avibactam[2]	2016/17–2018/19	3	x	148	4
Ceftobiprole	2011/12–2018/19	8	x	411	0.25‡
Ceftolozane/tazobactam[2]	2010/11–2018/19	9	x	447	≤0.015‡
Ciprofloxacin[3]	2008/09–2018/19	11	1	555	0.25‡
Colistin[1]	2010/11–2018/19	9	2	447	0.5
Gentamicin[1]	2008/09–2018/19	11	4	555	0.25‡
Imipenem	2008/09–2012/13, 2014/15–2018/19	10	4	503	0.12‡
Imipenem/relebactam[2,3]	2014/15–2018/19	5	2	262	0.12‡
Meropenem	2010/11, 2013/14–2018/19	7	8	350	0.25‡
Minocycline	2008/09–2012/13	5	x	241	≤0.06‡
Piperacillin/tazobactam[2]	2008/09–2018/19	11	x	555	0.03‡
Tetracycline	2008/09–2016/17	9	x	463	2‡
Tigecycline	2008/09–2012/13	5	x	241	0.25‡
Tobramycin[1]	2013/14–2018/19	6	4	314	0.25‡

81 isolates of non-ACB species are omitted; all modes were within ±1 doubling dilution of those for ACB.
‡ Most frequent MIC, but distribution was clearly bi- or multi-modal: refer to plot in Appendix.
[1] Epidemiological cut-off (ECOFF) 'breakpoint-in-brackets', intended to identify isolates with acquired resistance mechanisms likely to undermine effectiveness when used as a synergist in combination therapy.
[2] Tested with a fixed 4 mg/L concentration of the inhibitor (avibactam, relebactam or tazobactam).
[3] EUCAST notes that relebactam adds no clinical benefit because the OXA carbapenemases produced by *Acinetobacter* spp. are not inhibited by this agent.[5]

Table S7. *E. coli*: antibiotics analysed – years included, N of isolates, resistance breakpoints and mode MIC

Antimicrobial	Collection years included	N of years	Break-point R> mg/L	*E. coli* N of isolates	*E. coli* Mode MIC mg/L
Amikacin[1]	2013/14–2016/17	4	8	1006	1
Amoxicillin	2008/09–2018/19	11	8	2834	≥512‡
Co-amoxiclav[2]	2013/14–2018/19	6	8	1498	4‡
Cefotaxime	2008/09–2018/19	11	2	2834	0.06‡
Ceftazidime	2008/09–2018/19	11	4	2834	0.25‡
Ceftazidime/avibactam[3]	2016/17–2018/19	3	8	769	0.12
Ceftobiprole	2011/12–2018/19	8	0.25	1987	0.06‡
Ceftolozane/tazobactam [3]	2010/11–2018/19	9	2	2261	0.12
Cefuroxime	2008/09–2012/13	5	8	1336	4‡
Ciprofloxacin	2008/09–2018/19	11	0.5	2834	0.015‡
Colistin[1]	2010/11–2018/19	9	2	2261	0.5
Ertapenem	2014/15–2018/19	5	0.5	1243	0.015
Gentamicin[1]	2008/09–2018/19	11	2	2834	0.5‡
Imipenem	2008/09–2012/13; 2014/15–2018/19	10	4	2579	0.12
Imipenem/relebactam[3]	2014/15–2018/19	5	2	1243	0.12
Meropenem	2010/11;2013/14–2018/19	7	8	1772	0.015
Piperacillin/tazobactam[3]	2008/09–2018/19	11	8	2834	2‡
Tigecycline	2008/09–2012/13	5	0.5	1336	0.12
Tobramycin[1]	2013/14–2018/19	6	4	1498	0.5‡
Trimethoprim[4]	2014/15–2018/19	5	4	1243	0.25

‡ Most frequent MIC, but distribution was clearly bi- or multi-modal: refer to plot in Appendix.

1 Epidemiological cut-off (ECOFF) ‘breakpoint-in-brackets’, intended to identify isolates with acquired resistance mechanisms likely to undermine effectiveness when used as a synergist in combination therapy.

2 Tested with a fixed 2 mg/L concentration of clavulanate. (The 2:1 amoxicillin/clavulanate format, tested 2008/09–2012/13, has been abandoned and is not included in analyses of resistance.)

3 Tested with a fixed 4 mg/L concentration of the inhibitor (avibactam, relebactam or tazobactam).

4 Clinical breakpoint for uncomplicated urinary tract infection only. Trimethoprim is of limited relevance outside UTI, but co-trimoxazole is occasionally advocated for wider use in the NHS.[3,4]

Table S8. *Klebsiella* spp: antibiotics analysed – years included, N of isolates, resistance breakpoints and mode MIC

Antimicrobial	Collection years included	N of years	Break-point R > mg/L	*K. pneumoniae/ variicola*		*K. oxytoca*		*K. aerogenes*	
				N of isolates	Mode MIC mg/L	N of isolates	Mode MIC mg/L	N of isolates	Mode MIC mg/L
Amikacin[1]	2013/14–2016/17	4	8	640	1‡	248	1	112	1
Co-amoxiclav[2]	2013/14–2018/19	6	8	915	2‡	374	1‡	164	≥128
Cefotaxime	2008/09–2018/19	11	2	1656	0.06‡	698	0.03	335	0.12‡
Ceftazidime	2008/09–2018/19	11	4	1656	0.25‡	698	0.12	335	0.25‡
Ceftazidime/avibactam[3]	2016/17–2018/19	3	8	426	0.25	175	0.12	84	0.25
Ceftobiprole	2011/12–2018/19	8	0.25	1222	0.06‡	503	0.12‡	246	0.06
Ceftolozane/tazobactam[3]	2010/11–2018/19	9	2	1369	0.25‡	574	0.12	280	0.25
Cefuroxime	2008/09–2012/13	5	8, x[(4)]	741	2‡	324	2‡	171	4‡
Ciprofloxacin	2008/09–2018/19	11	0.5	1656	0.03‡	698	0.015‡	335	0.03‡
Colistin[1]	2010/11–2018/19	9	2	1369	0.5‡	574	0.5‡	280	0.5
Ertapenem	2014/15–2018/19	5	0.5	765	0.015‡	306	0.015	142	0.03‡
Gentamicin[1]	2008/09–2018/19	11	2	1656	0.5‡	698	0.5	335	0.5
Imipenem	2008/09–2012/13; 2014/15–2018/19	10	4	1506	0.12	630	0.12	313	0.5
Imipenem/relebactam[3]	2014/15–2018/19	5	2	765	0.5	306	0.25	142	0.25
Meropenem	2010/11;2013/14–2018/19	7	8	1062	≤0.03‡	445	≤0.03	198	≤0.03
Piperacillin/tazobactam[3]	2008/09–2018/19	11	8	1656	4‡	698	2‡	335	4
Tigecycline	2008/09–2012/13	5	x	741	0.5	324	0.25	171	0.25-0.5
Tobramycin[1]	2013/14–2018/19	6	2	915	0.5‡	374	0.5	164	0.25

‡ Most frequent MIC, but distribution was clearly bi- or multi-modal: refer to plot in Appendix.
x No breakpoint or epidemiological cut-off (ECOFF) available.
[1] ECOFF 'breakpoint-in-brackets', intended to identify isolates with acquired resistance mechanisms likely to undermine effectiveness when used as a synergist in combination therapy.
[2] Tested with a fixed 2 mg/L concentration of clavulanate. The 2:1 amoxicillin/clavulanate format, tested 2008/09–2012/13, has been abandoned and is not included in analyses of resistance.
[3] Tested with a fixed 4 mg/L concentration of the inhibitor (avibactam, relebactam or tazobactam).
[4] Cefuroxime breakpoint not relevant to *K. aerogenes*, which is inherently resistant.

Table S9. *E. cloacae* complex: antibiotics analysed – years included, N of isolates, resistance breakpoints and mode MIC

Antimicrobial	Collection years included	N of years	Break-point R > mg/L	*E. cloacae* complex N of isolates	*E. cloacae* complex Mode MIC mg/L
Amikacin[1]	2013/14–2016/17	4	8	319	1
Cefotaxime	2008/09–2018/19	11	2	968	0.12‡
Ceftazidime	2008/09–2018/19	11	4	968	0.25‡
Ceftazidime/avibactam[2]	2016/17–2018/19	3	8	220	0.25
Ceftobiprole	2011/12–2018/19	8	0.25	645	0.06‡
Ceftolozane/tazobactam[2]	2010/11–2018/19	9	2	750	0.25‡
Ciprofloxacin	2008/09–2018/19	11	0.5	968	0.015‡
Colistin[1]	2010/11–2018/19	9	2	750	0.5‡
Ertapenem	2014/15–2018/19	5	0.5	400	0.015‡
Gentamicin[1]	2008/09–2018/19	11	2	968	0.25‡
Imipenem	2008/09–2012/13; 2014/15–2018/19	10	4	900	0.25
Imipenem/relebactam[2]	2014/15–2018/19	5	2	400	0.25
Meropenem	2010/11; 2013/14–2018/19	7	8	573	0.03
Piperacillin/tazobactam[2]	2008/09–2018/19	11	8	968	2‡
Tigecycline	2008/09–2012/13	5	x	500	0.5
Tobramycin[1]	2013/14–2018/19	6	2	468	0.5‡

‡ Most frequent MIC, but distribution was clearly bi- or multi-modal: refer to plot in Appendix.
x No breakpoint or epidemiological cut-off (ECOFF) available.
[1] Epidemiological cut-off (ECOFF) 'breakpoint-in-brackets', intended to identify isolates with acquired resistance mechanisms likely to undermine effectiveness when used as a synergist in combination therapy,
[2] Tested with a fixed 4 mg/L concentration of the inhibitor (avibactam, relebactam or tazobactam).

Table S10. *Serratia* spp: antibiotics analysed – years included, N of isolates, resistance breakpoints and mode MIC

Antimicrobial	Collection years included	N of years	Break-point R > mg/L	*Serratia* spp.	
				N of isolates	Mode MIC mg/L
Amikacin[1]	2013/14–2016/17	4	8	273	2
Cefotaxime	2008/09–2018/19	11	2	759	0.25‡
Ceftazidime	2008/09–2018/19	11	4	759	0.25‡
Ceftazidime/avibactam[2]	2016/17–2018/19	3	8	227	0.25
Ceftobiprole	2011/12–2018/19	8	0.25	570	0.12‡
Ceftolozane/tazobactam[2]	2010/11–2018/19	9	2	648	0.5‡
Ciprofloxacin	2008/09–2018/19	11	0.5	759	0.06‡
Ertapenem	2014/15–2018/19	5	0.5	368	0.03
Gentamicin[1]	2008/09–2018/19	11	2	759	0.5‡
Imipenem	2008/09–2012/13; 2014/15–2018/19	10	4	704	0.5
Imipenem/relebactam[2]	2014/15–2018/19	5	2	368	0.25
Meropenem	2010/11; 2013/14–2018/19	7	8	501	0.06
Piperacillin/tazobactam[2]	2008/09–2018/19	11	8	759	2‡
Tigecycline	2008/09–2012/13	5	x	336	0.5
Tobramycin[1]	2013/14–2018/19	6	8	423	2

Results include 689 isolates of *S. marcescens*, 47 *S. liquefaciens* and 23 of other or unidentified *Serratia* species.

‡ Most frequent MIC, but distribution was clearly bi- or multi-modal, often related to species mix: refer to plots in Appendix.

x No breakpoint or epidemiological cut-off (ECOFF) available.

[1] Epidemiological cut-off (ECOFF) 'breakpoint-in-brackets', intended to identify isolates with acquired resistance mechanisms likely to undermine effectiveness when used as a synergist in combination therapy.

[2] Tested with a fixed 4 mg/L concentration of the inhibitor (avibactam, relebactam or tazobactam).

Table S11. Proteeae: antibiotics analysed – years included, N of isolates, resistance breakpoints and mode MIC

Antimicrobial	Collection years included	N of years	Break-point R > mg/L	*P. mirabilis*		*M. morganii*	
				N of isolates	Mode MIC mg/L	N of isolates	Mode MIC mg/L
Amikacin[1a]	2013/14–2016/17	4	16	144	2	27	2[††]
Amoxicillin[2]	2008/09–2018/19	11	8	354	0.5[‡]	73	256
Co-amoxiclav[2]	2013/14–2018/19	6	8	207	0.5[‡]	44	≥128
Cefotaxime	2008/09–2018/19	11	2	354	≤0.008[‡]	73	0.015[‡]
Ceftazidime	2008/09–2018/19	11	4	354	0.03[‡]	73	0.06[‡]
Ceftazidime/avibactam[3]	2016/17–2018/19	3	8	99	0.03	25	0.03[††]
Ceftobiprole	2011/12–2018/19	8	0.25	299	0.03	57	0.06
Ceftolozane/tazobactam[3]	2010/11–2018/19	9	2	327	0.12	63	0.12
Cefuroxime[2]	2008/09–2012/13	5	8	147	1[‡]	29	32[††]
Ciprofloxacin	2008/09–2018/19	11	0.5	354	0.03[‡]	73	0.015[‡]
Ertapenem	2014/15–2018/19	5	0.5	173	0.008	37	0.015
Gentamicin[1b]	2008/09–2018/19	11	4	354	0.5	73	0.5[‡]
Imipenem	2008/09–2012/13;2014/15–2018/19	10	4	320	2[‡]	66	2
Imipenem/relebactam[3]	2014/15–2018/19	5	x	173	2	37	1-2
Meropenem	2010/11;2013/14–2018/19	7	8	235	0.06	50	0.06
Piperacillin/tazobactam[3]	2008/09–2018/19	11	8	354	0.25	73	0.12
Tigecycline	2008/09–2012/13	5	x	147	2	29[‡‡]	0.5
Tobramycin[1c]	2013/14–2018/19	6	4	207	1[‡]	44	1

Not included: 13 isolates of other species of *Proteus* (not *mirabilis*) and 5 of *Providencia*.

[‡] Most frequent MIC, but distribution was clearly bi- or multi-modal: refer to plot in Appendix (*P. mirabilis* only).

[††] Caution: based on fewer than 30 isolates.

x No breakpoint or epidemiological cut-off (ECOFF) available.

[1] Epidemiological cut-off (ECOFF) 'breakpoint-in-brackets', intended to identify isolates with acquired resistance mechanisms likely to undermine effectiveness when used as a synergist in combination therapy. EUCAST guidance is to use the ECOFF given at www.mic.eucast.org if this differs from the "best-fit" value shown in brackets in the table of clinical breakpoints. The table shows the ECOFFs for *P. mirabilis*; values used for other species/genera are noted below.

[1a] Amikacin: using "best-fit" ECOFF (R >8 mg/L) for all except *P. mirabilis*.

[1b] Gentamicin: using "best-fit" ECOFF (R >2 mg/L) for all except *P. mirabilis* (R >4 mg/L) and *Providencia* (no ECOFF or breakpoint listed).

[1c] Tobramycin: using *P. mirabilis* ECOFF for *Morganella* and "best-fit" (R >2 mg/L) for other species of *Proteus*. No ECOFF or breakpoint for *Providencia*.

[2] Amoxicillin is of interest only for *P. mirabilis* and co-amoxiclav of interest only against *P. mirabilis* and *P. vulgaris*. Co-amoxiclav was tested with a fixed 2 mg/L concentration of clavulanate. The 2:1 amoxicillin/clavulanate format, tested 2008/09–2012/13, has been abandoned and is not included in analyses of resistance.
[3] Tested with a fixed 4 mg/L concentration of the inhibitor (avibactam, relebactam or tazobactam).

Table S12. *Citrobacter* spp. and less frequent Enterobacterales: antibiotics analysed – N of years included, N of isolates, resistance breakpoints and mode MIC

Antimicrobial	N[1] of years	Break-point R> mg/L	*C. koseri*		*C. freundii*		*R. ornithinolytica*		*R. terrigena*		*H. alvei*	
			N of isolates	Mode MIC mg/L	N of isolates	Mode MIC mg/L	N of isolates	Mode MIC mg/L	N of isolates	Mode MIC mg/L	N of isolates	Mode MIC mg/L
Amikacin[2]	4	8	107	1	35	1	14	xx	2	xx	16	1
Amoxicillin	11	8	210	128	122	≥512‡	51	64	19	32‡	37	64
Co-amoxiclav[3]	6	8	153	1	64	≥128	25	1‡	3	xx	21	≥128
Cefotaxime	11	2	210	0.06‡	122	0.12‡	51	0.03	19	0.03‡	37	0.5‡
Ceftazidime	11	4	210	0.12	122	0.25‡	51	0.12	19	0.12‡	37	2‡
Ceftazidime/avibactam[4]	3	8	76	0.06	41	0.12	16	0.12	1	xx	8	xx
Ceftobiprole	8	0.25	183	0.06‡	93	0.06‡	43	0.06	3	xx	29	0.12‡
Ceftolozane/tazobactam	9	2	197	0.12	103	0.12	48	0.12	3	xx	32	0.25‡
Cefuroxime[5]	5	8	57	4	58	2‡	26	1	16	2‡	16	4
Ciprofloxacin	11	0.5	210	≤0.008	122	0.015	51	0.015	19	0.015‡	37	0.015
Colistin[2]	9	2	197	0.5	103	0.5	48	0.5	3	xx	32	8‡
Ertapenem	5	0.5	133	0.015	57	0.008‡	24	0.015	3	xx	16	0.03-0.12
Gentamicin[2]	11	2	210	0.25	122	0.25	51	0.25	19	0.25	37	0.25
Imipenem	10	4	190	0.12	115	0.5	50	0.12	19	0.12	32	0.25‡
Imipenem/relebactam	5	2	133	0.12	57	0.25	24	0.5	3	xx	16	0.12
Meropenem	7	8	167	0.015	74	0.03	30	0.03	3	xx	24	0.03
Piperacillin/tazobactam	11	8	210	2	122	2	51	2	19	2	37	8
Tigecycline[6]	5	0.5	57	0.25	58	0.25	26	0.25	16	0.25	16	0.5
Tobramycin[2]	6	2	153	0.5	64	0.5‡	25	0.5	3	xx	21	0.25

Not included: 23 isolates of other species of *Citrobacter*; 11 of other or unknown species of *Raoultella* (10 *R. planticola*); 15 from six other genera.

Caution: modes based on small numbers. All for *Raoultella* and *Hafnia* species and some for *C. freundii* based on <50 isolates; **‡‡** <30 isolates; **xx** <15 isolates, not shown.

‡ Most frequent MIC, but distribution was clearly bi- or multi-modal: refer to plot in Appendix (*Citrobacter* only).

[1] Number of years when each agent was tested; actual years are listed in Table S3. These infrequent species were not all found in all the yearly collections.

[2] Epidemiological cut-off (ECOFF) 'breakpoint-in-brackets', intended to identify isolates with acquired resistance mechanisms likely to undermine effectiveness when used as a synergist in combination therapy.

[3] Co-amoxiclav – to which *C. freundii* and *H. alvei* are inherently resistant – was tested with a fixed 2 mg/L concentration of clavulanate. The 2:1 amoxicillin/clavulanate format, tested 2008/09–2012/13, has been abandoned and is not included in analyses of resistance.

[4] Tested with a fixed 4 mg/L concentration of the inhibitor (avibactam, relebactam or tazobactam).

[5] Cefuroxime breakpoint applies to *Raoultella* only in this table. [6] Tigecycline breakpoint applies to *C. koseri* only in this table.

BSAC hospital-acquired LRTI resistance surveillance – patient characteristics

Sex

In general, there was a preponderance of male patients, ranging from 59% (*Pseudomonas*) to 71% (*Klebsiella* spp.) and 77% (Citrobacter spp.) with little clear trend over time.
There were also clear differences between species within organism groups.

Table S13. Proportion (%) of male patients by organism species

Organism	N[1]	Male, %
MSSA	1,851	60.6
MRSA	482	62.0
S. aureus, all	*2,333*	*60.9*
P. aeruginosa	2,332	59.0
Pseudomonas: other/spp.	20	xx
Pseudomonas, all	*2,352*	*59.1*
Acinetobacter – ACB complex	552	63.6
Acinetobacter – non-ACB species	81	51.9
Acinetobacter, all	*633*	*62.1*
Enterobacterales		
E. coli	2,834	66.8
K. pneumoniae/variicola	1,656	69.5
K. oxytoca	698	70.5
K. aerogenes	335	81.5
Klebsiella, all	*2,689*	*71.3*
E. cloacae complex	968	64.9
S. marcescens	687	67.5
S. liquefaciens	47	64††
Serratia: other or unnamed	23	xx
Serratia, all	*757*	*66.6*
P. mirabilis	353	60.9
Proteus: other or unnamed	13	xx
M. morganii	73	67.1
Providencia spp.	5	xx
Proteeae, all	*444*	*62.8*
C. koseri	210	81.9
C. freundii	122	69.7
Citrobacter: other or unnamed	23	xx
Citrobacter, all	*355*	*77.2*
Raoultella spp.	81	66.7
Hafnia alvei	37	57††
Very infrequent genera	15	xx

[1] Number of isolates with sex data for the source patient. (Missing: 10/13508; ≤0.1%)
†† Caution: ≤50 isolates; imprecise estimates.
xx ≤30 isolates; % not shown

Age

All organism groups had infants aged under one year old as a distinct subgroup of source patients, largest for *Acinetobacter* (13%) and *Enterobacter* (12%) and ≤6% for all other groups. Within organism groups, the proportion of infants was higher among patients with LRTI caused by MSSA (7%, vs <1% for MRSA) and those with *K. oxytoca* (9% infants, versus 5% for *K. pneumoniae* and 4% for *K. aerogenes*). The proportions of patients aged ≥80 years were in the range 14–20% for all organism groups except *Acinetobacter* (6%).

Table S14. Patient age: summary measures by organism

		Quartiles, years			Isolates in age group shown, %		
Organism	N[1]	Q1	Q2	Q3	<1 year	≥65 years	≥80 years
MSSA	1,848	36	58	73	7.3	39.9	12.3
MRSA	482	56	69	78	0.8	59.8	20.5
P. aeruginosa	2,332	51	67	76	2.9	55.0	16.2
ACB complex	554	23	52	68	13.4	32.3	5.8
Acinetobacter other	81	16	57	71	13.6	39.5	6.2
E. coli	2,829	54	68	77	5.3	57.9	19.5
K. pneumoniae/variicola	1,652	55	68	77	4.9	57.1	17.6
K. oxytoca	697	52	67	77	9.0	55.5	17.9
K. aerogenes	334	55	69	76	3.9	61.1	15.9
E. cloacae complex	966	44	64	75	12.3	49.0	15.0
S. marcescens	687	51	68	76	4.7	58.7	16.6
S. liquefaciens	47††	62	68	76	4.3	70.2	10.6
P. mirabilis	352	60	70	77	2.0	64.8	16.5
M. morganii	73	60	67	77	4.1	60.3	17.8
C. koseri	210	57	67.5	78	2.9	63.3	20.5
C. freundii	122	60	68.5	78	0.0	66.4	20.5
Raoultella spp.	80	57	68	76.5	7.5	61.2	15.0

[1] Number of isolates with age data for the source patient. (Missing: 26/13508; 0.2%)
†† Caution: ≤50 isolates; imprecise estimates.
Not shown: organisms of other or unnamed species, each with data for ≤40 isolates (20 *Pseudomonas*, 23 *Serratia*; 13 *Proteus*, 5 *Providencia*, 37 *Hafnia*, 15 other infrequent genera of Enterobacterales).

Patients aged ≥80 years are an older subset of those who are ≥65 years old.

Figure S1. Patient age: histograms and trends by organism group

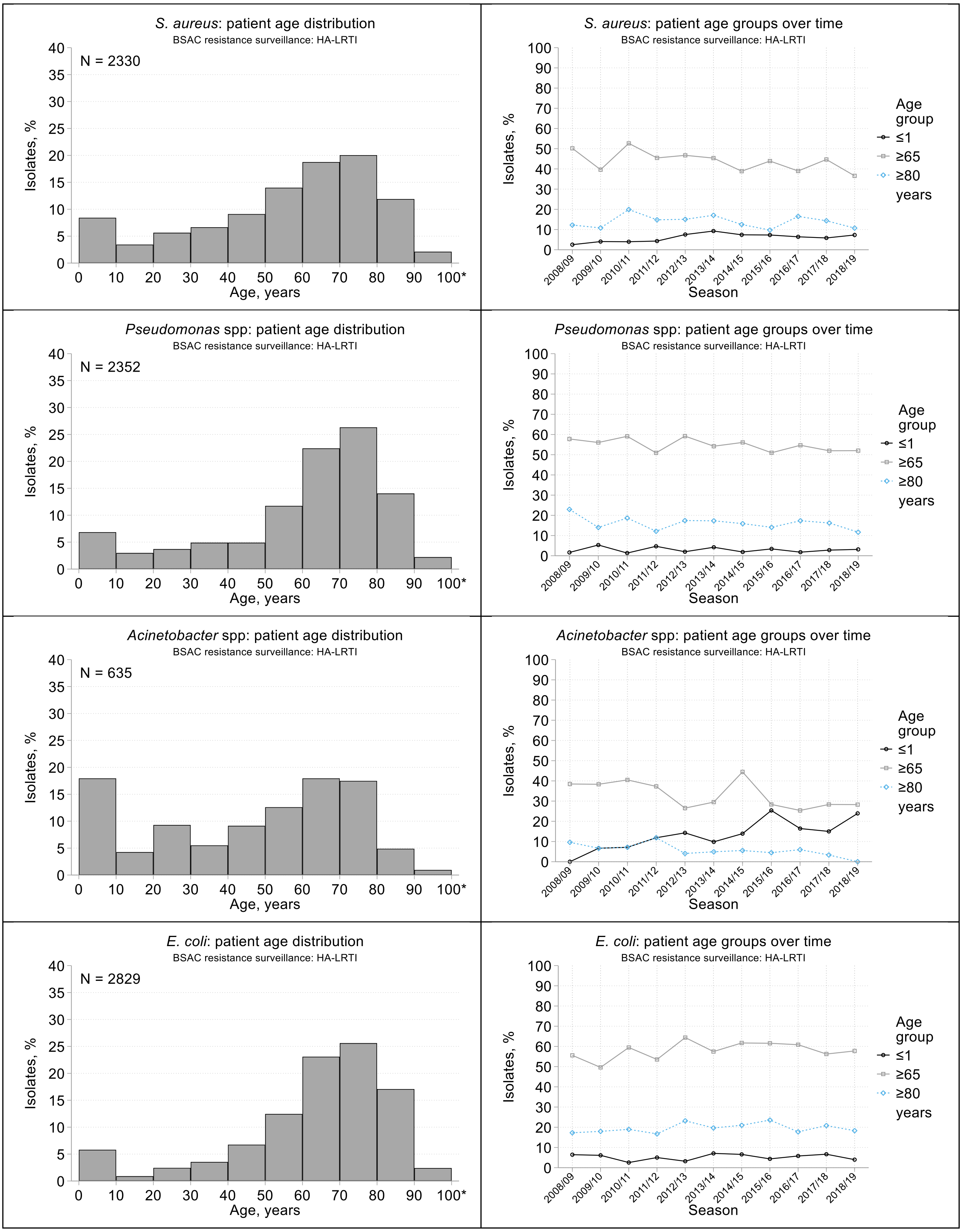

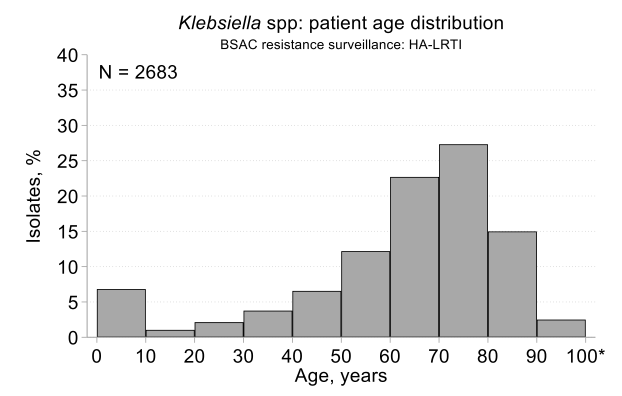

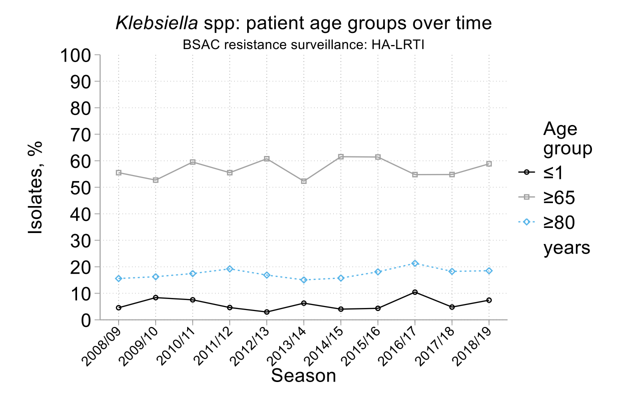

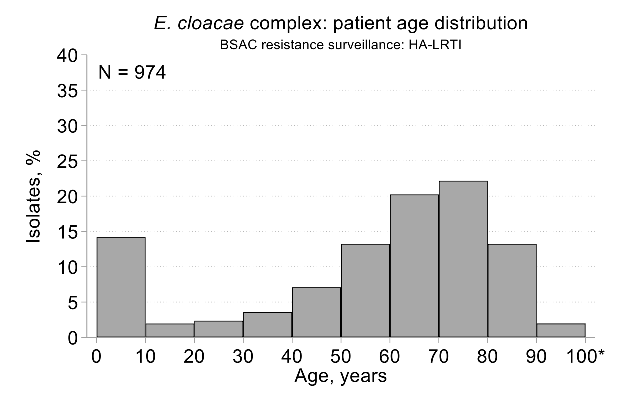

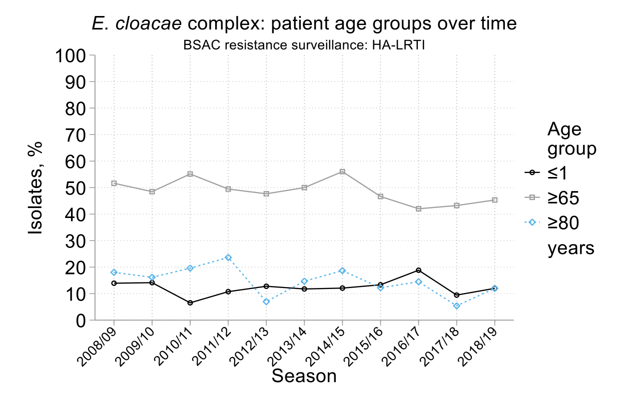

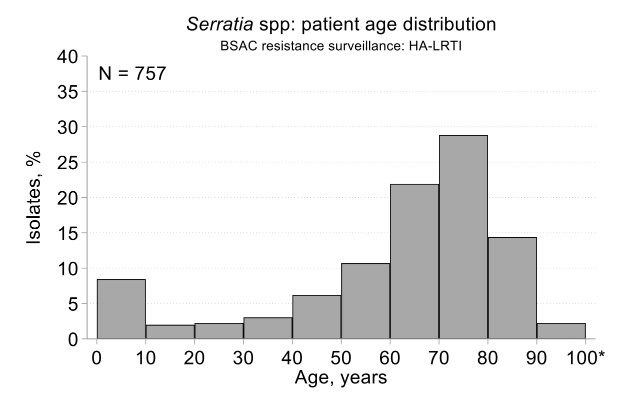

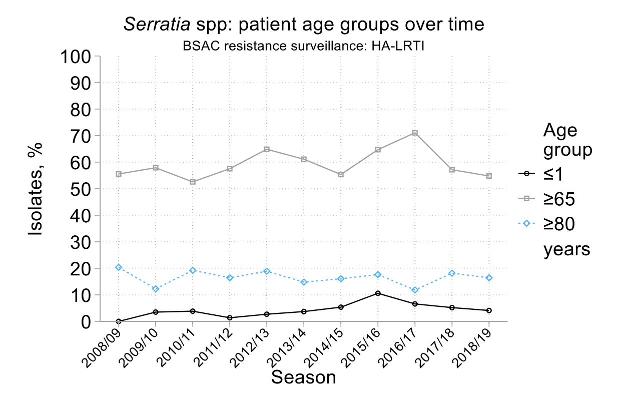

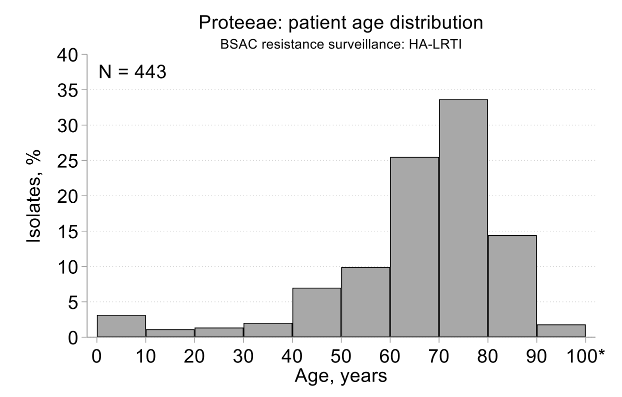

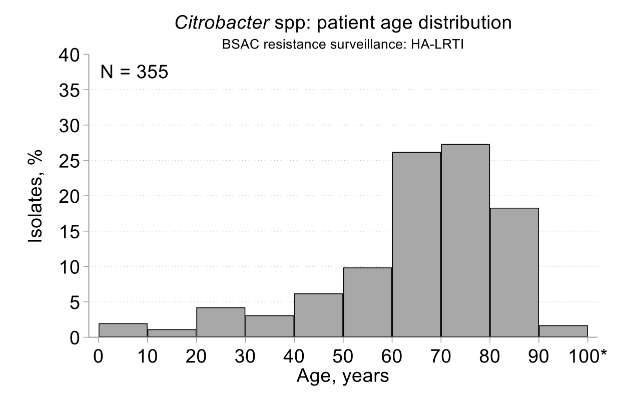

* The final bar of histograms includes all patients aged ≥90 years.
Age group trends over time not shown for Proteeae, *Citrobacter* and less frequent genera of Enterobacterales as isolates are few (mean <50/year).

Care setting: intensive/critical care speciality (ICU)

Samples were from patients in hospital for >48 hours, as per protocol, with >99.9% compliance. Six isolates were included despite missing data for care setting, and one was non-compliant (source patient in hospital, but for ≤48 hours).

Table S15. Proportion (%) of isolates from ICU patients, by organism

Organism	N[1]	ICU, %
MSSA	1,852	42.5
MRSA	482	25.2
S. aureus, all		
P. aeruginosa	2,335	33.7
Pseudomonas: other/spp.	20	xx
Pseudomonas, all		
Acinetobacter – ACB complex	555	51.3
Acinetobacter – non-ACB species	81	53.1
Acinetobacter, all		
Enterobacterales		
E. coli	2,834	40.6
K. pneumoniae/variicola	1,656	40.5
K. oxytoca	698	45.6
K. aerogenes	335	52.3
Klebsiella, all		
E. cloacae complex	968	44.8
S. marcescens	689	40.3
S. liquefaciens	47	42††
Serratia: other or unnamed	23	xx
Serratia, all		
P. mirabilis	354	39.5
Proteus: other or unnamed	73	52.2
M. morganii	13	xx
Providencia spp.	5	xx
Proteeae, all		
C. koseri	210	50.2
C. freundii	122	44.1
Citrobacter: other or unnamed	23	xx
Citrobacter, all		
Raoultella spp.	81	43.0
Hafnia alvei	37	52††
Enterobacterales of very infrequent genera	15	xx

[1] Number of isolates with speciality/ICU data for the source patient. (Missing hospital speciality data: 418/13508; 3.1%.)

†† Caution: ≤50 isolates; imprecise estimates.

xx ≤30 isolates; % not shown

Figure S2. Trends in proportion of isolates from ICU patients, by organism group

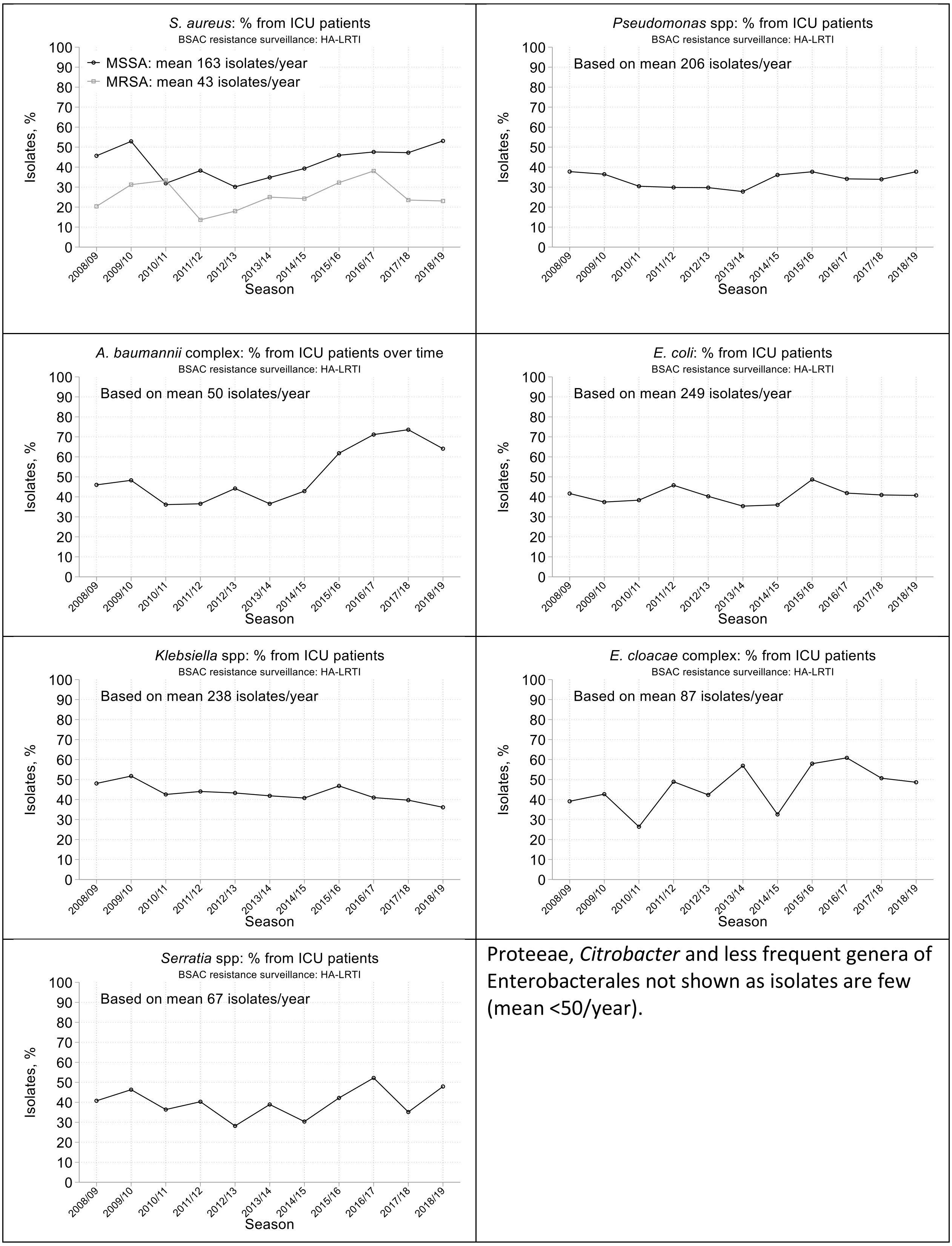

Proteeae, *Citrobacter* and less frequent genera of Enterobacterales not shown as isolates are few (mean <50/year).

Specimen type

The most frequent specimen type was sputum, ranging from 60% (*Acinetobacter*) to 78% (*Pseudomonas*, Proteeae) of isolates. Tracheal/endotracheal specimens and bronchoalveolar lavage accounted for approximately equal shares of the remainder, with just 1–3% from other specimen types.

Table S16. Specimen types – % by organism

Organism group	N[1]	Specimen type, %			
		Sputum	Tracheal[2]	BAL[3]	Other[4,5]
S. aureus	2,330	74.1	13.0	10.7	2.2
Pseudomonas	2,352	78.0	11.5	9.5	0.9
Acinetobacter	636	59.9	23.6	13.8	2.7
E. coli	2,833	76.8	11.2	10.3	1.7
Klebsiella	2,687	74.7	11.4	12.4	1.6
Enterobacter	968	69.0	16.4	12.5	2.1
Serratia	759	73.4	13.3	12.0	1.3
Proteeae	445	78.2	12.1	8.3	1.3
Citrobacter	355	74.6	14.6	9.0	1.7
Other Enterobacterales	133	63.2	18.0	17.3	1.5

[1] Number of isolates with data for specimen type. (Missing: 10/13508; <0.1%)
[2] Tracheal/endotracheal secretions/aspirates/tips.
[3] Bronchoalveolar lavage.
[4] The 224 isolates from 'Other' specimen types included 36 from upper respiratory tract samples such as nasopharyngeal secretions/aspirates (0.3% of all isolates),
[5] The 'Other' LRTI specimen types recorded for the remaining 188 isolates were predominantly bronchial washings/aspirates and chest drains.

References

1. Allen M, Reynolds R, Mushtaq S *et al*. The British Society for Antimicrobial Chemotherapy Resistance Surveillance Project: methods and limitations. *J Antimicrob Chemother* 2025; **80** (Suppl 4): iv7–iv21.

2. Brady C, Cleenwerck I, Venter S, et al. Taxonomic evaluation of the genus *Enterobacter* based on multilocus sequence analysis (MLSA): proposal to reclassify *E. nimipressuralis* and *E. amnigenus* into *Lelliottia gen. nov*. as *Lelliottia nimipressuralis comb. nov*. and L*elliottia amnigena comb. nov.*, respectively, *E. gergoviae* and *E. pyrinus i*nto *Pluralibacter gen. nov.* as *Pluralibacter gergoviae comb. nov*. and *Pluralibacter pyrinus comb. nov.*, respectively, *E. cowanii, E. radicincitans, E. oryzae and E. arachidis* into *Kosakonia gen. nov.* as *Kosakonia cowanii comb. nov., Kosakonia radicincitans comb. nov., Kosakonia oryzae comb. nov*. and *Kosakonia arachidis comb. nov.*, respectively, and *E. turicensis, E. helveticus* and *E. pulveris* into *Cronobacter* as *Cronobacter zurichensis nom. nov., Cronobacter helveticus comb. nov.* and *Cronobacter pulveris comb. nov.*, respectively, and emended description of the genera Enterobacter and Cronobacter. *Syst Appl Microbiol* 2013; **36**: 309–19.

3. Anon. Antimicrobial-specific guidance. *Scott Antimicrob Prescr Group*. Available at: https://www.sapg.scot/guidance-qi-tools/antimicrobial-specific-guidance/.

4. Barton E, Macgowan AP. Use of intravenous co-trimoxazole to treat bacterial infection: analysis of 50 treatment episodes. *J Chemother Florence Italy* 2010; **22**: 267–9.

5. Anon. eucast: Clinical breakpoints and dosing of antibiotics. Available at: https://www.eucast.org/clinical_breakpoints/.

APPENDIX – MIC distributions

BSAC hospital-acquired LRTI resistance surveillance

These graphs are presented as thumbnails for reasons of space. Please zoom in to read.

The red vertical lines show EUCAST v12.0 (2022) breakpoint(s)[5] or, if used for analysis in the absence of breakpoints, ECOFFs – see tables S4–S12. Where two lines are shown, they indicate the susceptible (S ≤) and resistant (R >) breakpoints; MICs between these bounds are designated I "susceptible, increased exposure". More commonly, there is a single line because the S and R breakpoints are coincident and there is no I category. Occasionally, noted below, there is no S category, and the single line demarcates R from I.

Some distributions were affected by excessive censoring due to testing of restricted concentration ranges, usually in earlier years. These years' data are omitted, as noted in the affected plots, to show the true range more accurately.

Collection years and number of isolates are noted within each plot. The MIC axes all span ≤0.001 to ≥1024 mg/L, with labelled values showing the range of MICs actually observed in those years.

Combinations lacking S category
S. aureus: ciprofloxacin
Pseudomonas: ceftazidime, ciprofloxacin, imipenem, piperacillin/tazobactam
Acinetobacter: ciprofloxacin
Enterobacterales: cefuroxime (all); imipenem (Proteeae)

S. aureus – *MSSA & MRSA*

MSSA

Ceftaroline

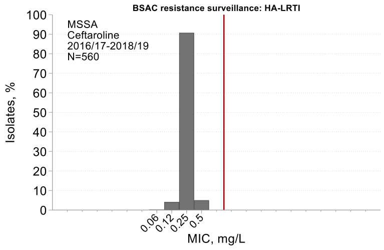

Ceftobiprole

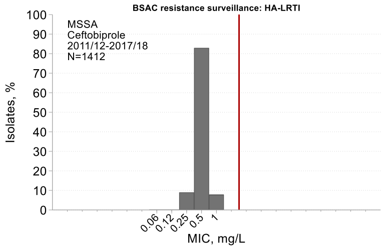

Ciprofloxacin

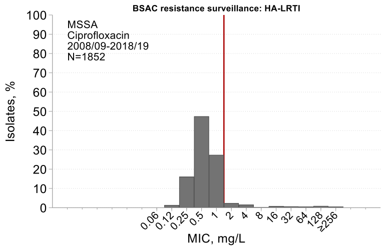

Clindamycin

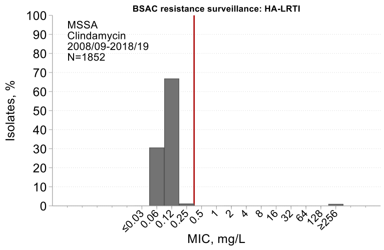

Erythromycin

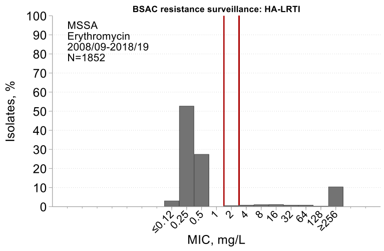

Fusidic acid

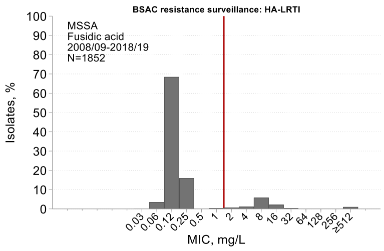

MRSA

Ceftaroline

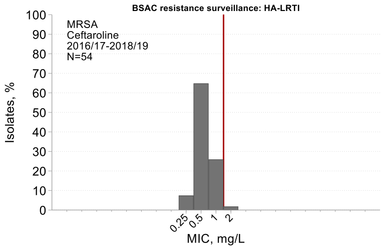

Ceftobiprole

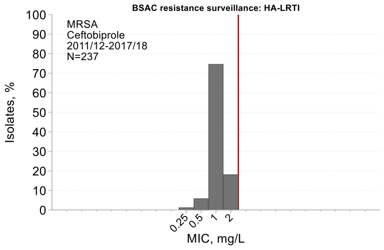

Ciprofloxacin

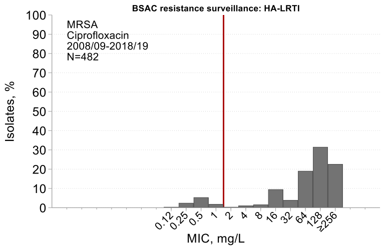

Clindamycin

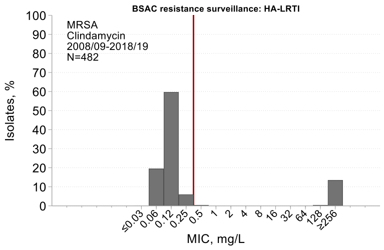

Erythromycin

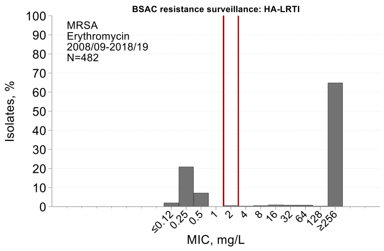

Fusidic acid

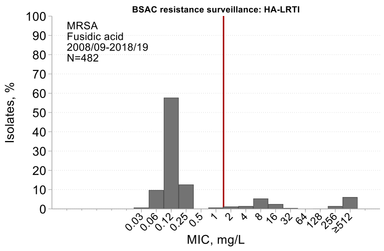

MSSA	MRSA

Gentamicin (MSSA)

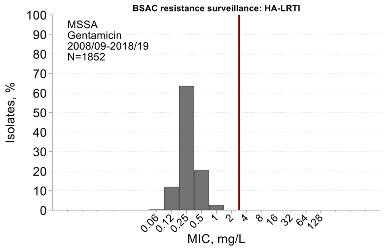

Gentamicin (MRSA)

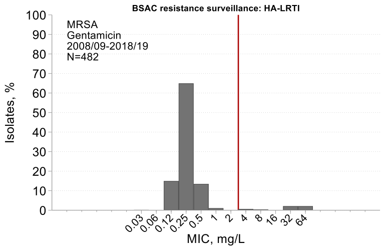

Linezolid (MSSA)

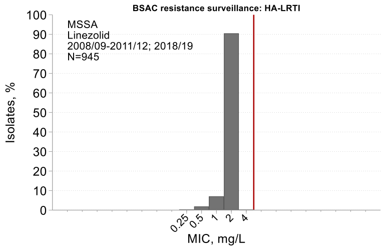

Linezolid (MRSA)

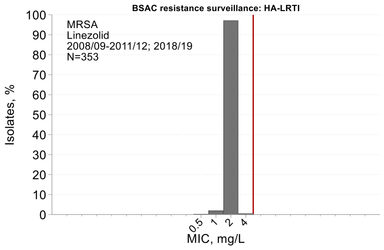

Minocycline (MSSA)

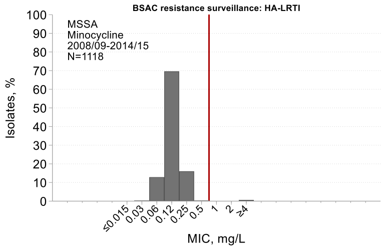

Minocycline (MRSA)

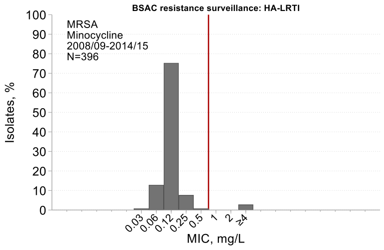

Mupirocin (MSSA)

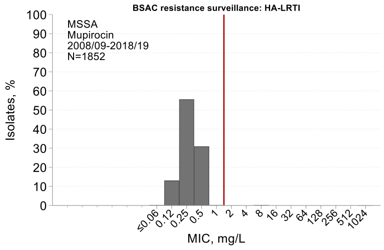

Mupirocin (MRSA)

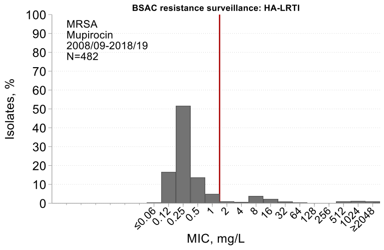

Oxacillin (MSSA)

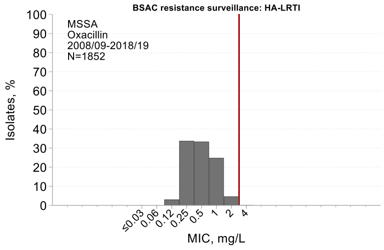

Oxacillin (MRSA)

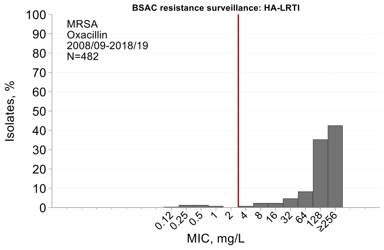

Penicillin (MSSA)

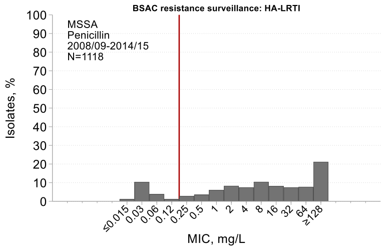

Penicillin (MRSA)

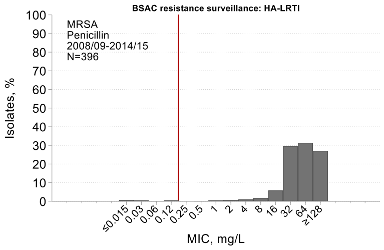

MSSA	MRSA

Rifampicin (MSSA)

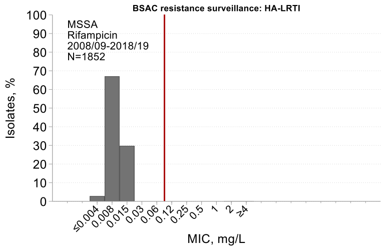

Rifampicin (MRSA)

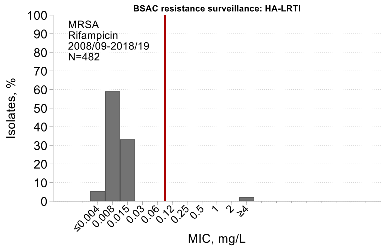

Tedizolid (MSSA)

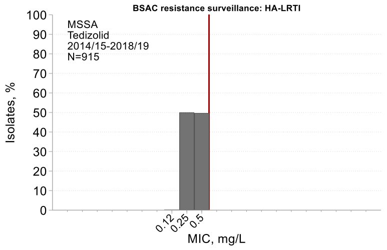

Tedizolid (MRSA)

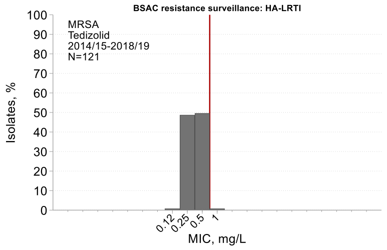

Teicoplanin (MSSA)

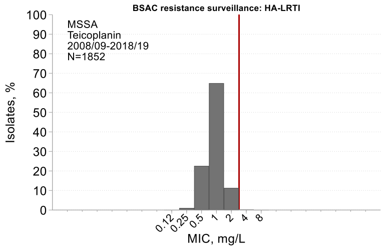

Teicoplanin (MRSA)

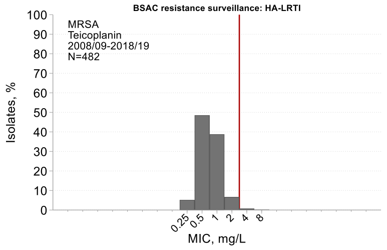

Tetracycline (MSSA)

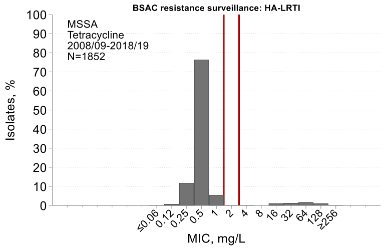

Tetracycline (MRSA)

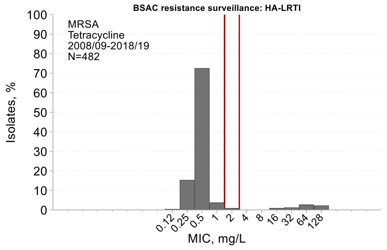

Tigecycline (MSSA)

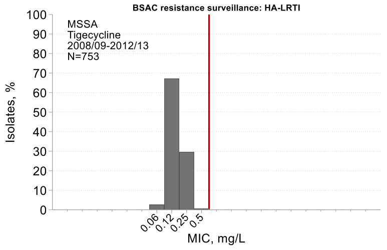

Tigecycline (MRSA)

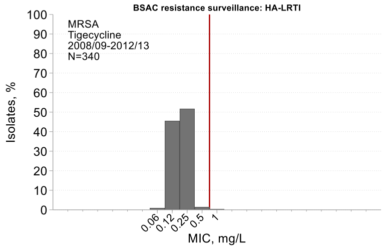

Trimethoprim (MSSA)

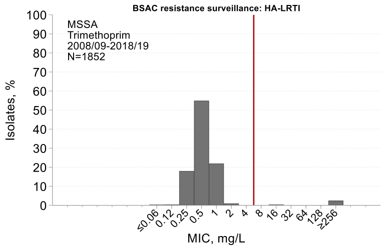

Trimethoprim (MRSA)

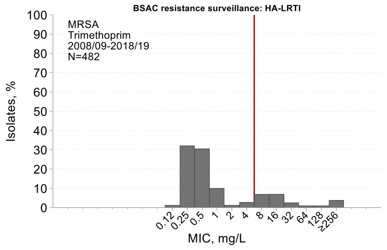

MSSA	MRSA
**Vancomycin**	**Vancomycin**

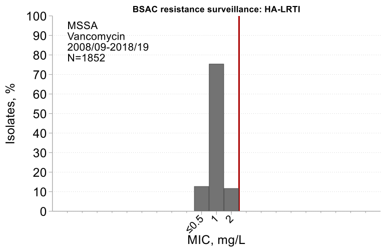

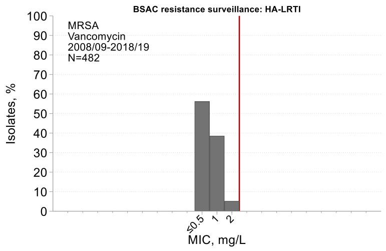

P. aeruginosa

Pseudomonas aeruginosa

Amikacin

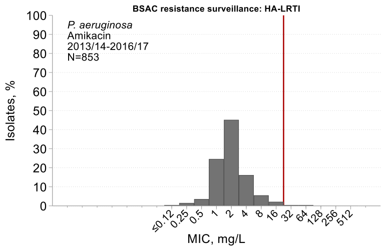

Ceftazidime

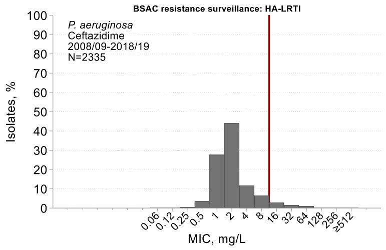

Ceftazidime/avibactam

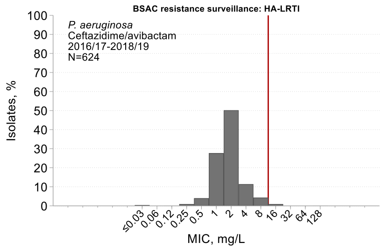

Ceftobiprole

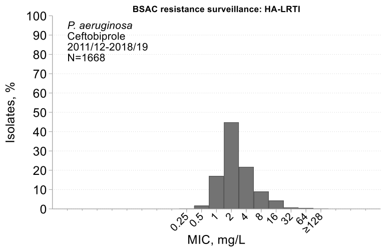

Ceftolozane/tazobactam

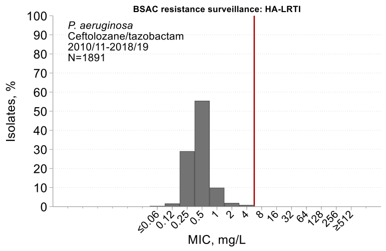

Ciprofloxacin

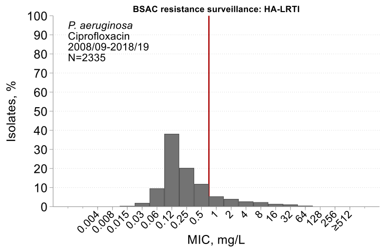

Colistin

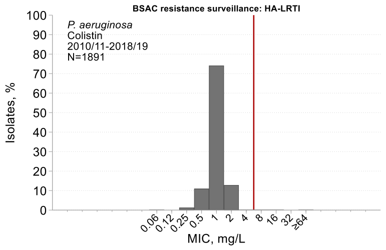

Gentamicin

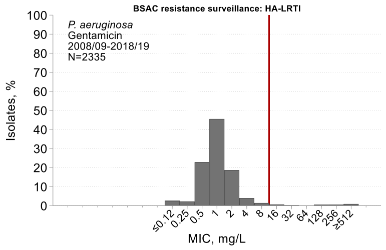

Imipenem

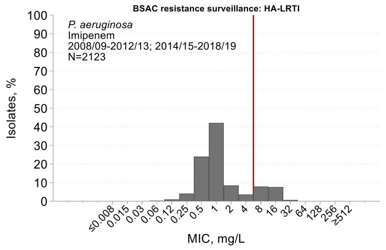

Imipenem/relebactam

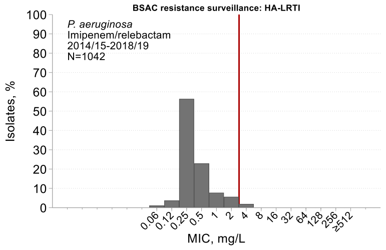

Meropenem

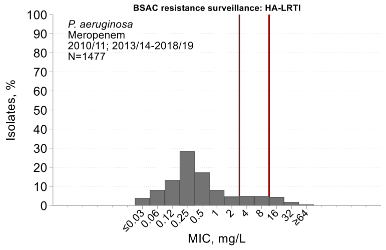

Piperacillin/tazobactam

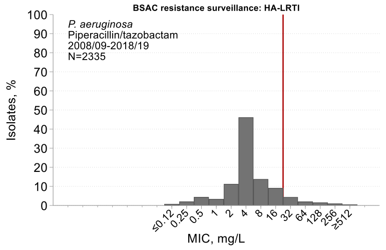

Tobramycin

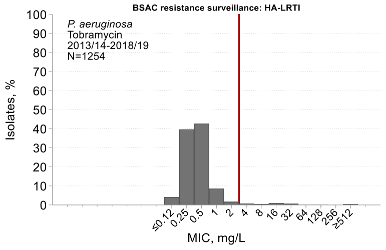

Acinetobacter calcoaceticus-baumannii (ACB) complex

Acinetobacter calcoaceticus-baumannii (ACB) complex

Amikacin

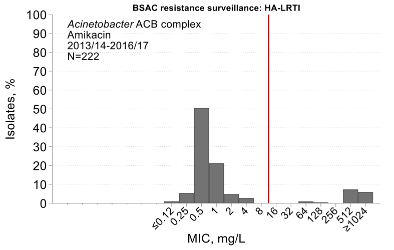

Ceftazidime

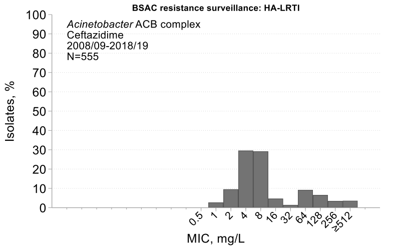

Ceftazidime/avibactam

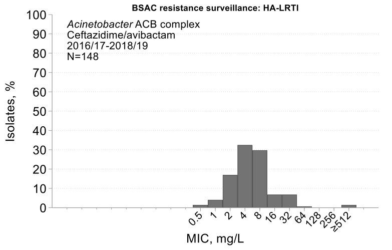

Ceftobiprole

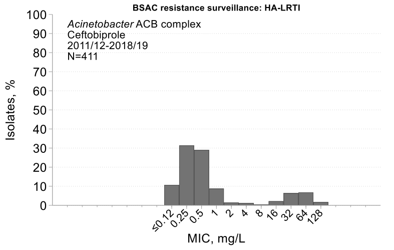

Ceftolozane/tazobactam

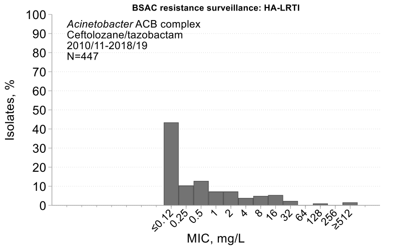

Ciprofloxacin

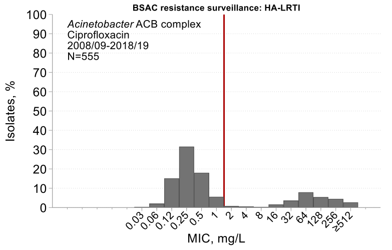

Colistin

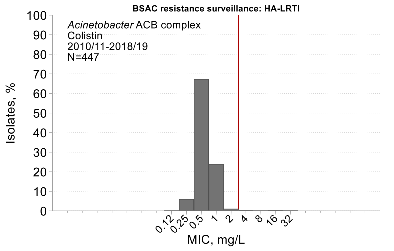

Gentamicin

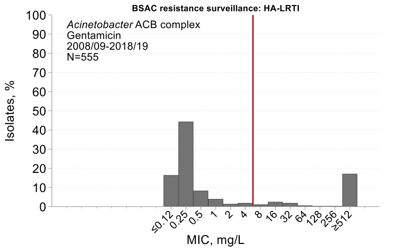

Imipenem

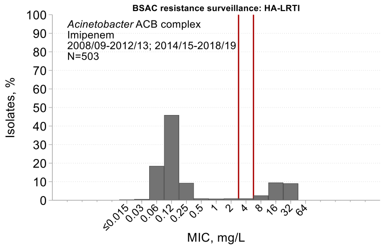

Imipenem/relebactam

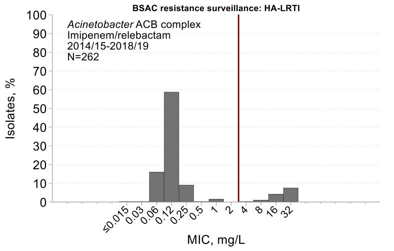

Meropenem

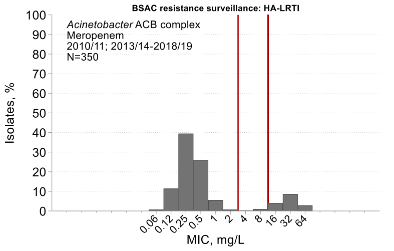

Minocycline

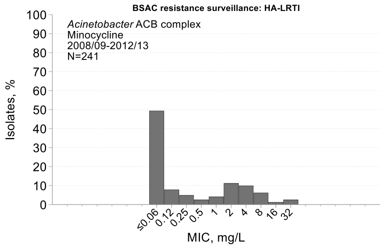

Piperacillin/tazobactam

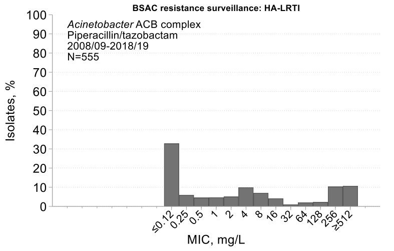

Tetracycline

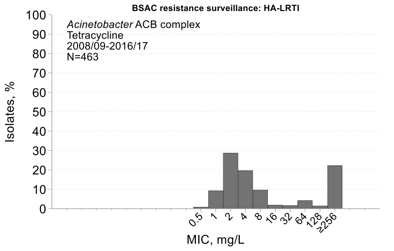

Tigecycline

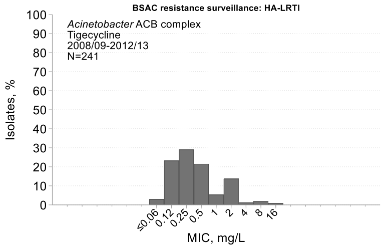

Tobramycin

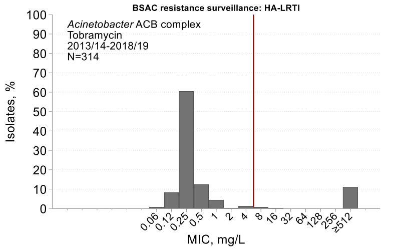

E. coli

Escherichia coli

Amikacin

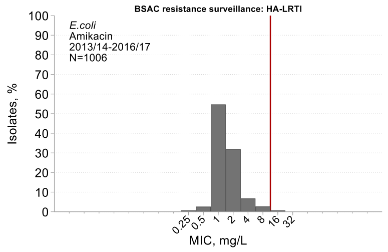

Amoxicillin

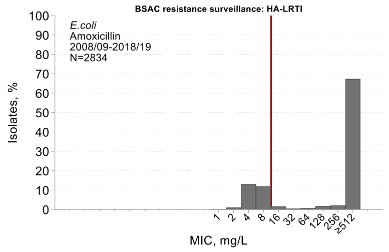

Co-amoxiclav (2:1 ratio)

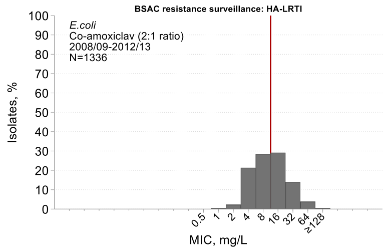

Co-amoxiclav (2 mg/L clavulanate)

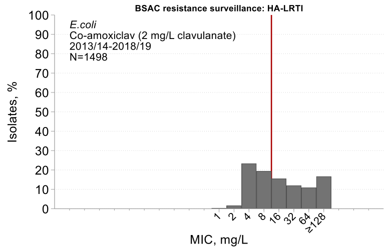

Cefotaxime

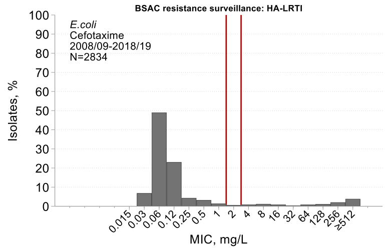

Ceftazidime

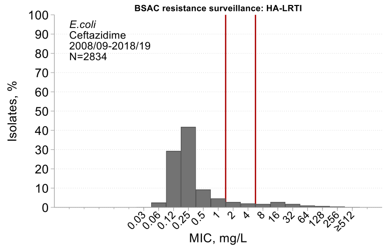

Ceftazidime/avibactam

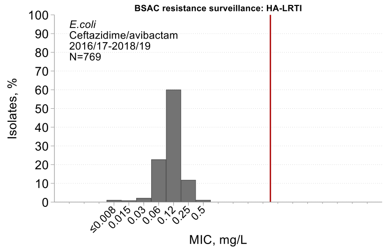

Ceftobiprole

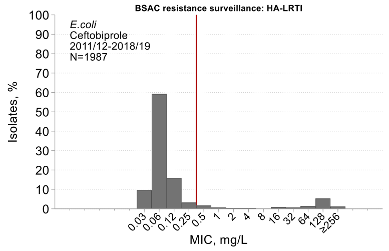

Ceftolozane/tazobactam

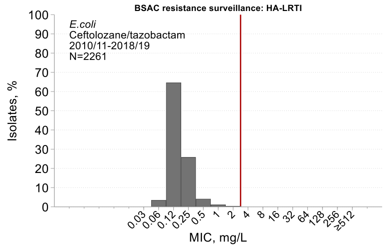

Cefuroxime

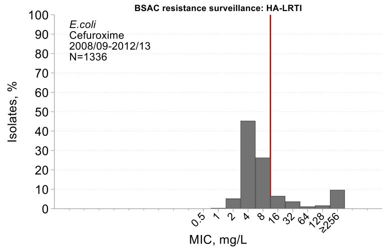

Ciprofloxacin

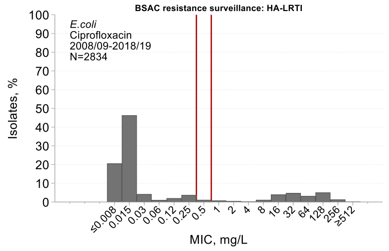

Colistin

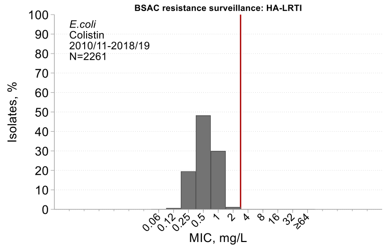

Ertapenem

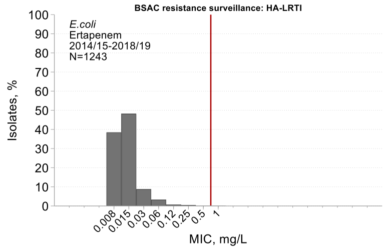

Gentamicin

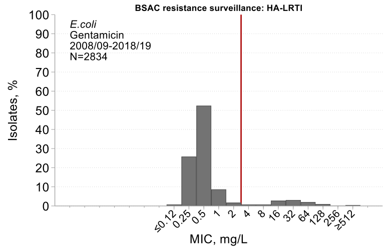

Imipenem

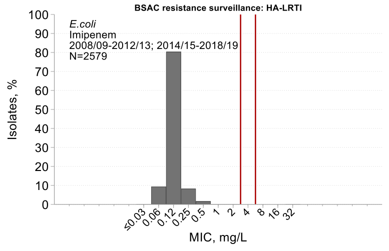

Imipenem/relebactam

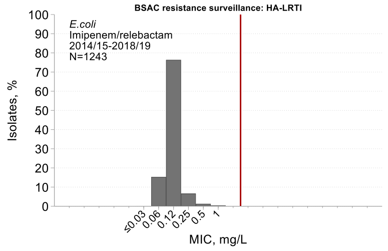

Meropenem

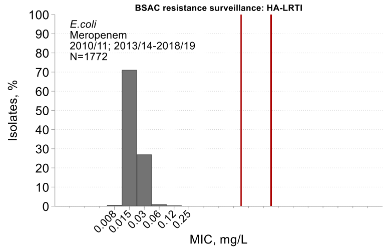

Piperacillin/tazobactam

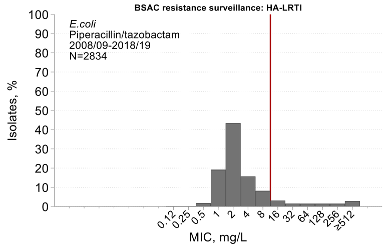

Escherichia coli

Tigecycline

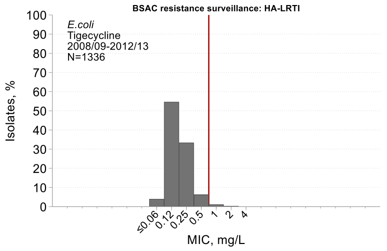

Tobramycin

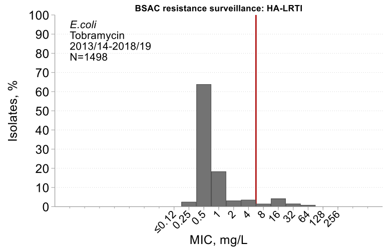

Trimethoprim

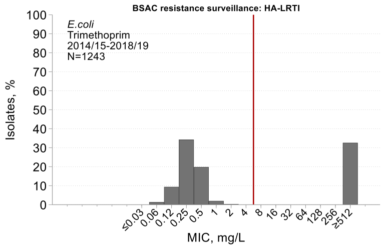

Klebsiella spp.

K. pneumoniae/ variicola

Amikacin

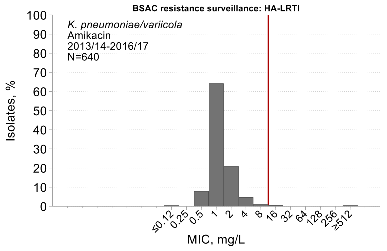

Co-amoxiclav (2:1 ratio)

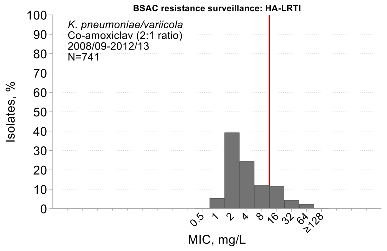

Co-amoxiclav (2 mg/L clavulanate)

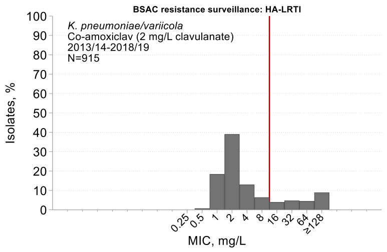

Cefotaxime

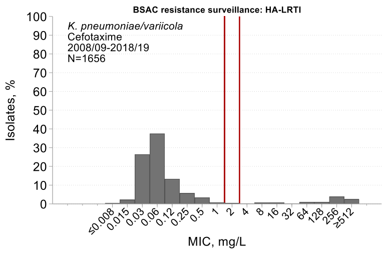

K. oxytoca

Amikacin

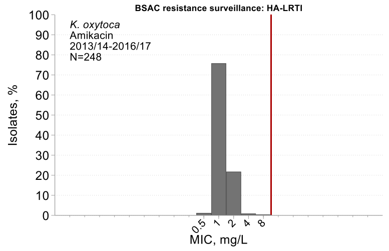

Co-amoxiclav (2:1 ratio)

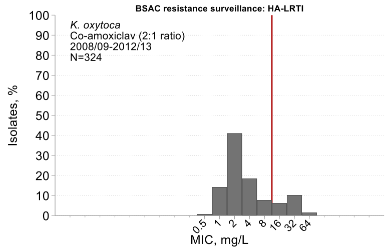

Co-amoxiclav (2 mg/L clavulanate)

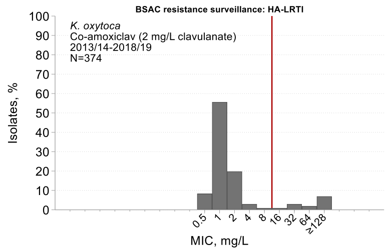

Cefotaxime

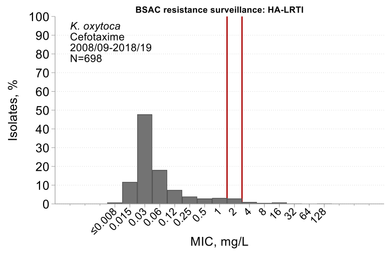

K. aerogenes

Amikacin

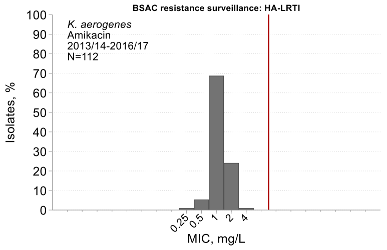

Co-amoxiclav (2:1 ratio)

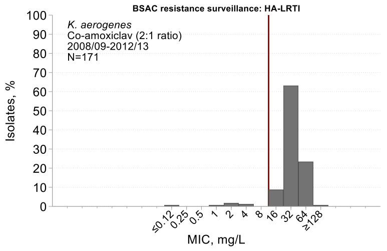

Co-amoxiclav (2 mg/L clavulanate)

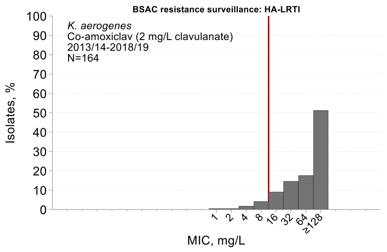

Cefotaxime

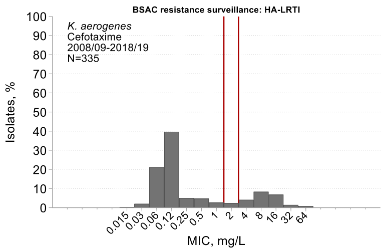

K. pneumoniae/ variicola	*K. oxytoca*	*K. aerogenes*

Ceftazidime

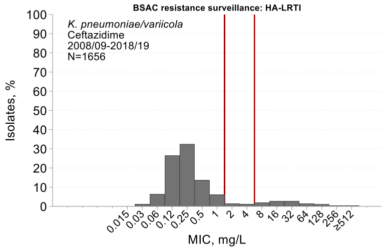

Ceftazidime

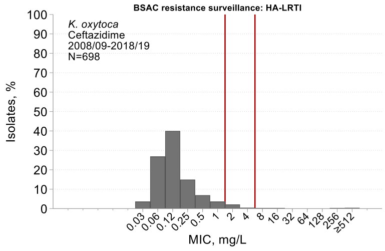

Ceftazidime

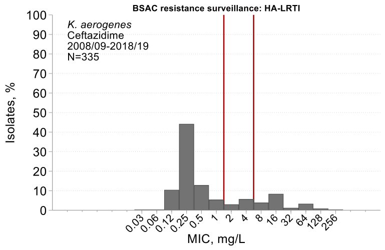

Ceftazidime/avibactam

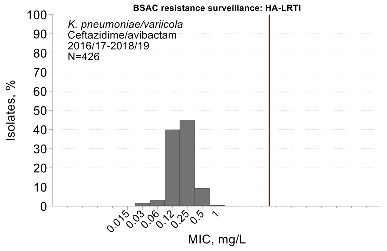

Ceftazidime/avibactam

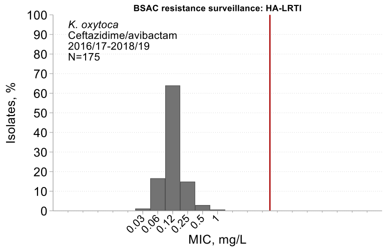

Ceftazidime/avibactam

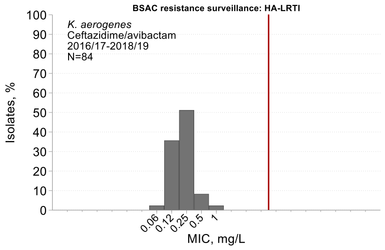

Ceftobiprole

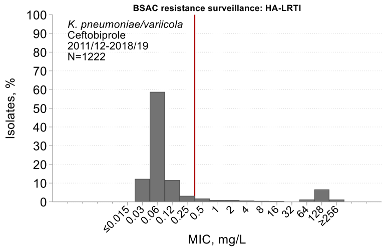

Ceftobiprole

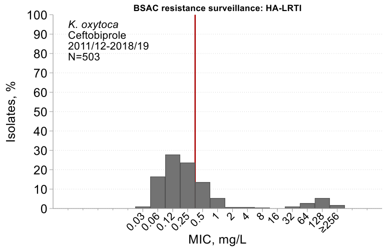

Ceftobiprole

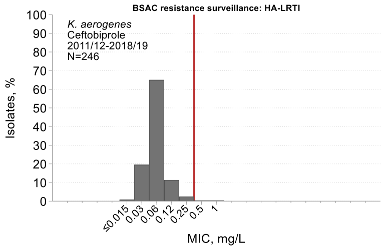

Ceftolozane/tazobactam

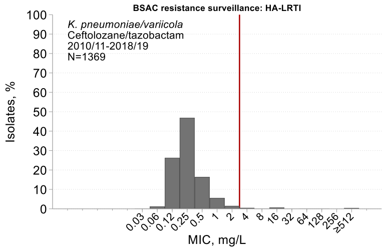

Ceftolozane/tazobactam

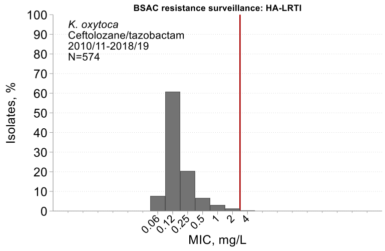

Ceftolozane/tazobactam

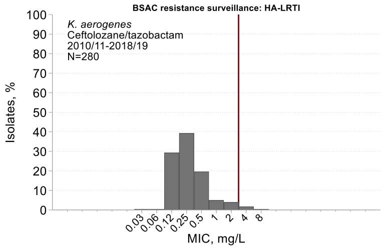

Cefuroxime

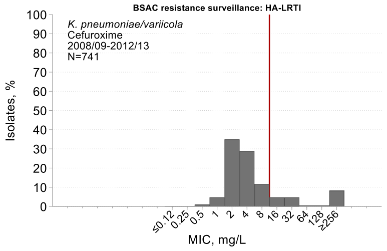

Cefuroxime

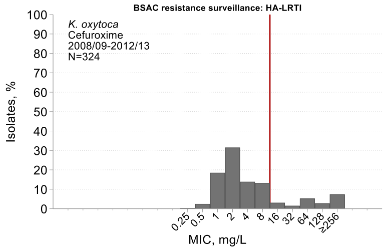

Cefuroxime

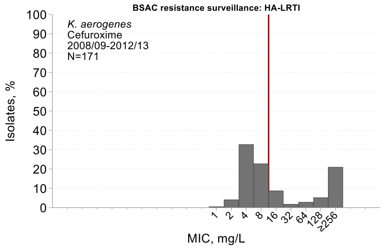

Ciprofloxacin

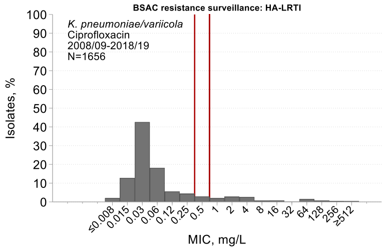

Ciprofloxacin

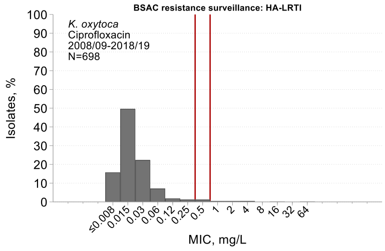

Ciprofloxacin

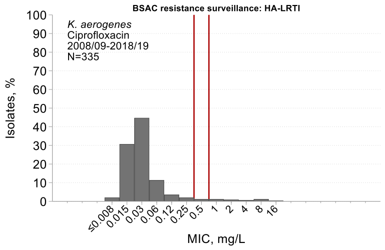

K. pneumoniae/ variicola	*K. oxytoca*	*K. aerogenes*

Colistin

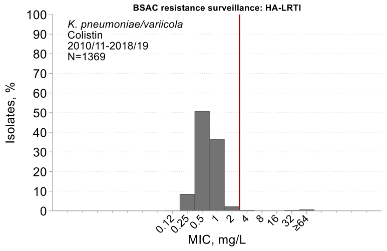

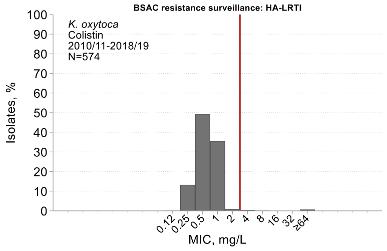

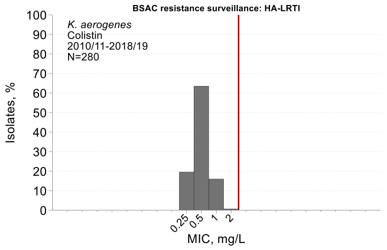

Ertapenem

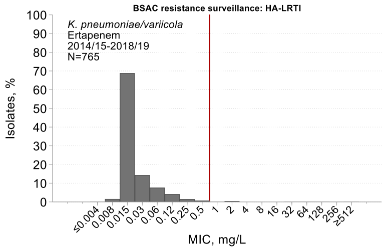

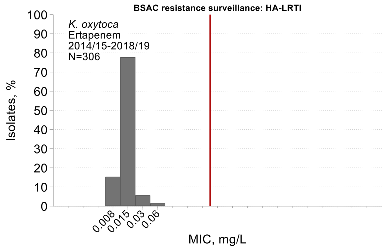

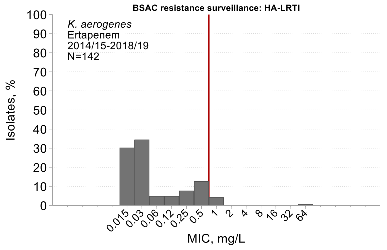

Gentamicin

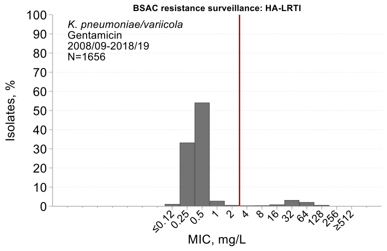

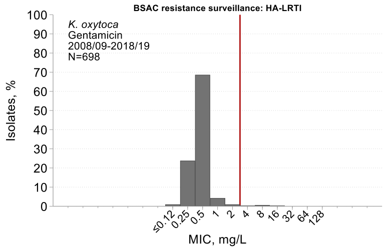

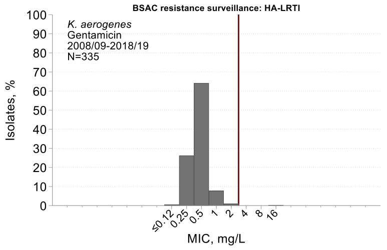

Imipenem

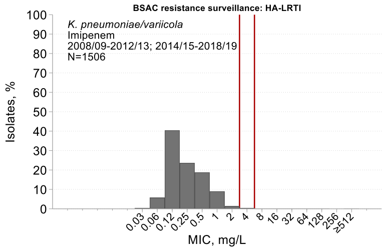

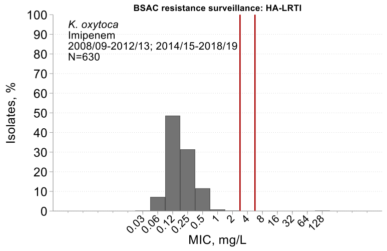

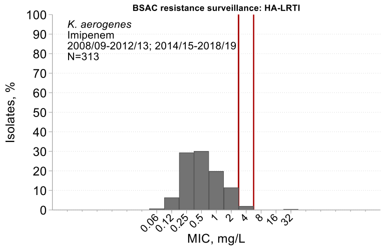

Imipenem/relebactam

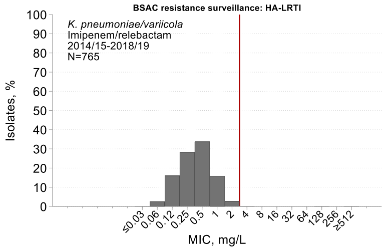

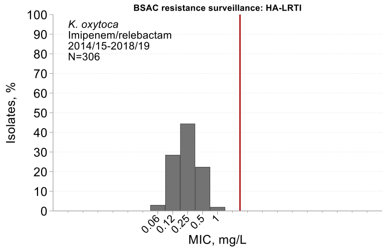

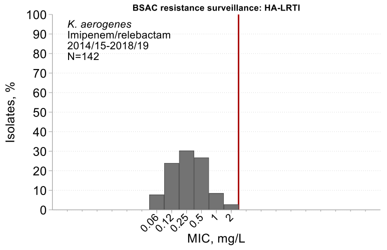

Meropenem

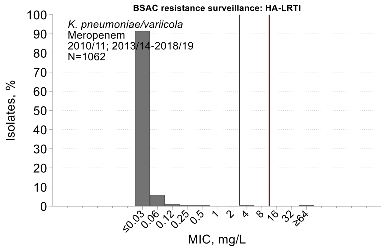

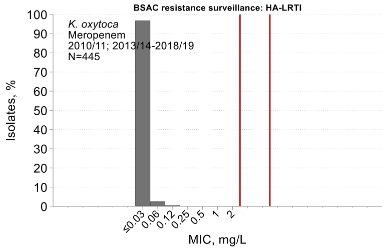

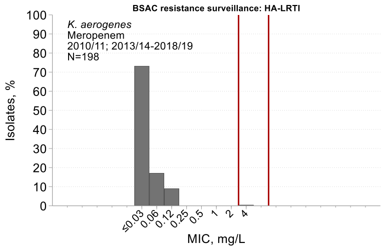

K. pneumoniae/ variicola

Piperacillin/tazobactam

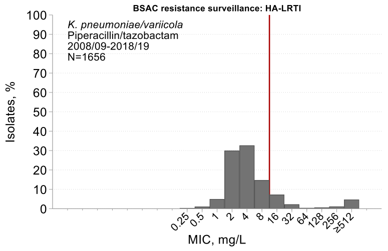

Tigecycline

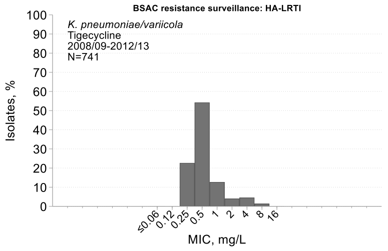

Tobramycin

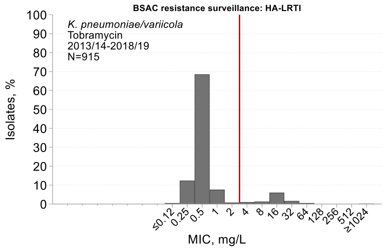

K. oxytoca

Piperacillin/tazobactam

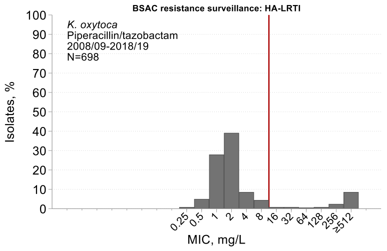

Tigecycline

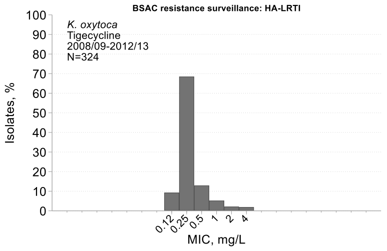

Tobramycin

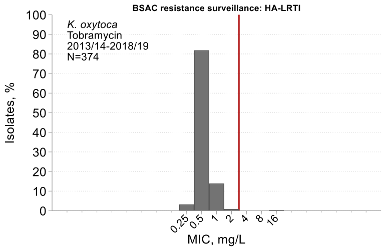

K. aerogenes

Piperacillin/tazobactam

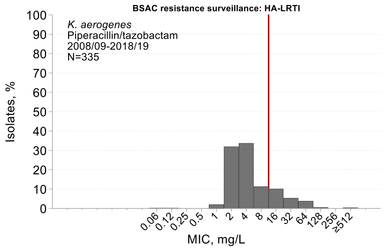

Tigecycline

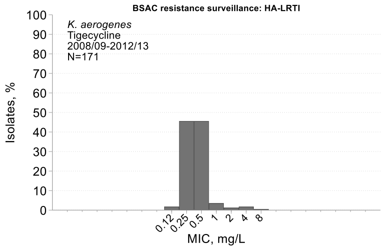

Tobramycin

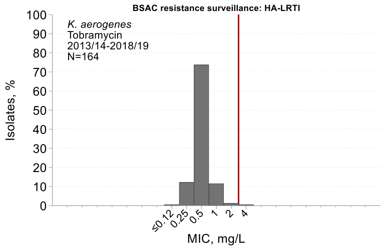

E. cloacae complex

Enterobacter cloacae complex

Amikacin

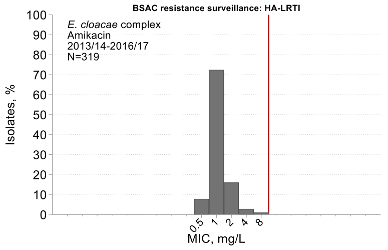

Cefotaxime

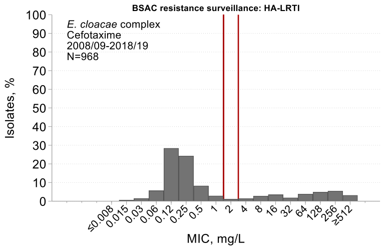

Ceftazidime

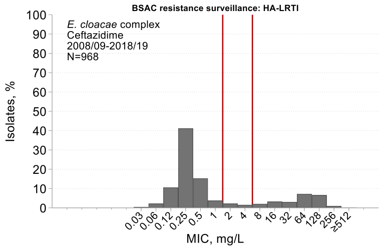

Ceftazidime/avibactam

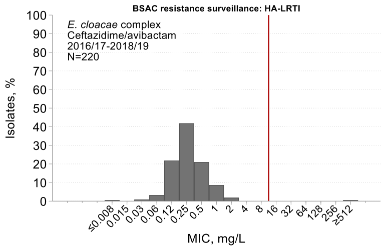

Ceftobiprole

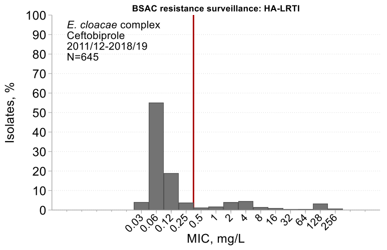

Ceftolozane/tazobactam

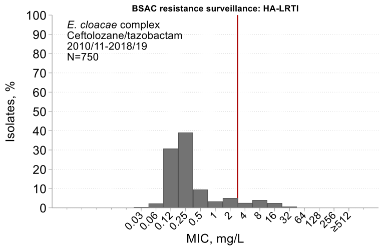

Enterobacter cloacae complex

Ciprofloxacin

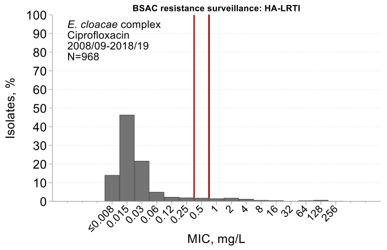

Colistin

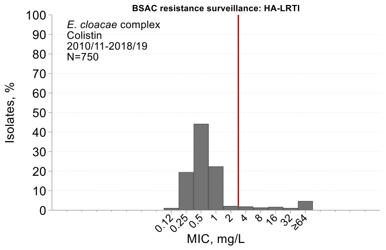

Ertapenem

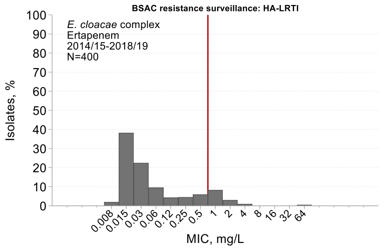

Gentamicin

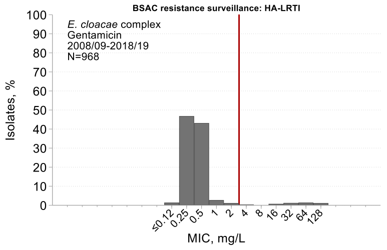

Imipenem

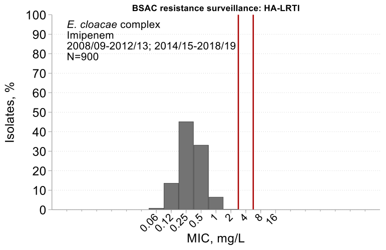

Imipenem/relebactam

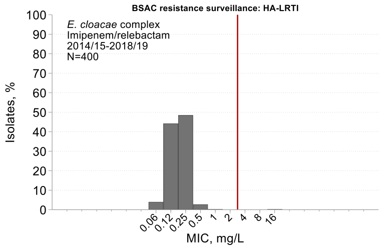

Meropenem

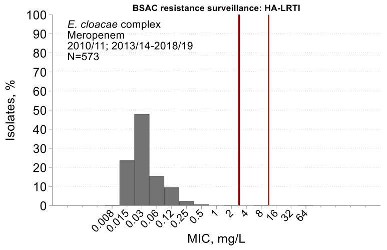

Piperacillin/tazobactam

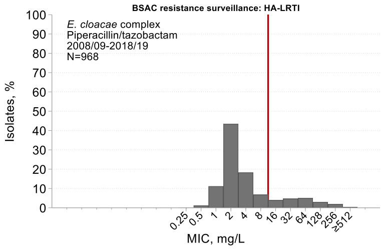

Tigecycline

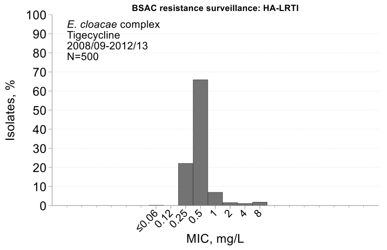

Tobramycin

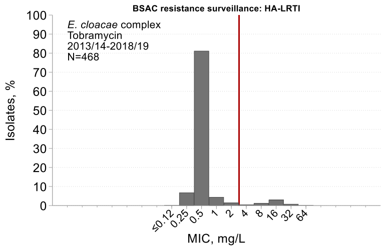

Serratia

Distributions for *S. marcescens* are shown for all antimicrobials. Those for *S. liquefaciens* are limited to agents tested in at least 10 of the 11 seasons' surveillance, including 43–47 isolates.

Serratia marcescens

Amikacin

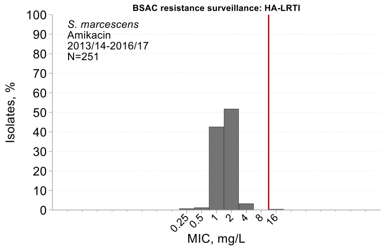

Cefotaxime

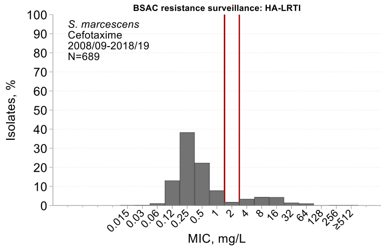

Ceftazidime

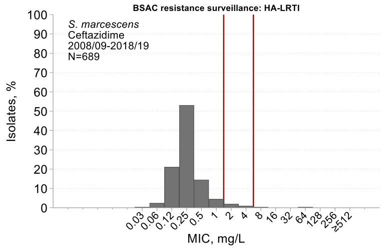

Ceftazidime/avibactam

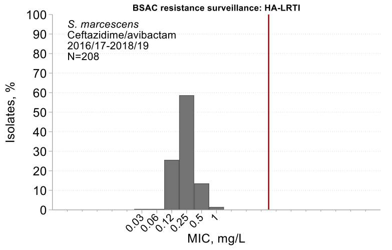

Ceftobiprole

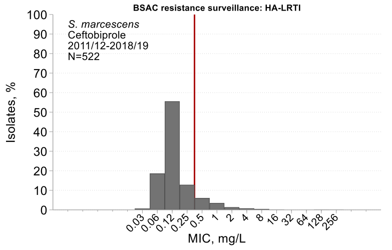

Ceftolozane/tazobactam

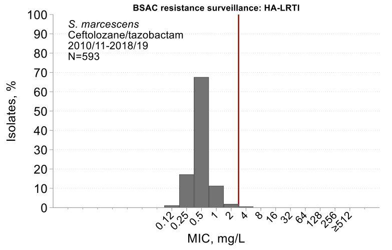

Ciprofloxacin

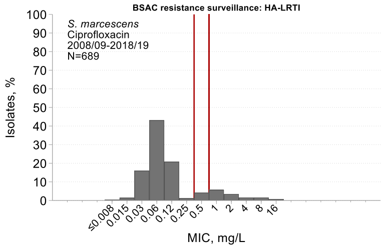

Ertapenem

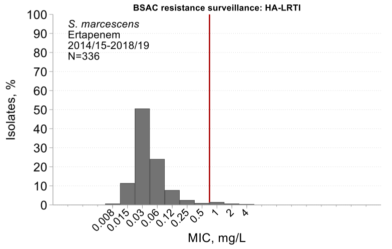

Gentamicin

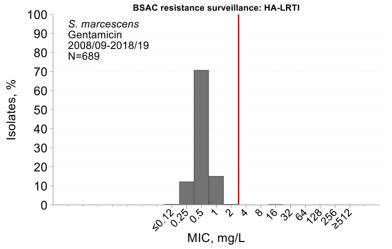

Imipenem

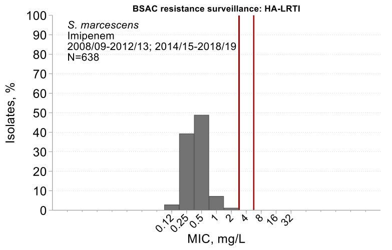

Imipenem/relebactam

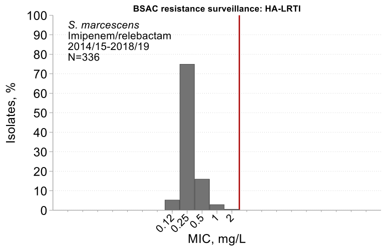

Meropenem

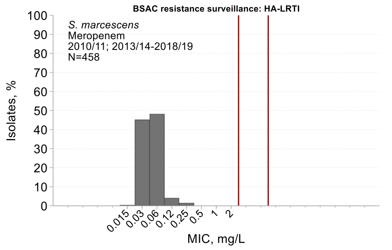

Piperacillin/tazobactam

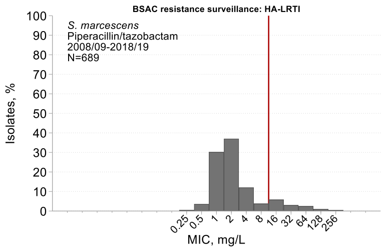

Tigecycline

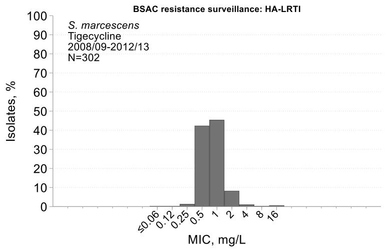

Tobramycin

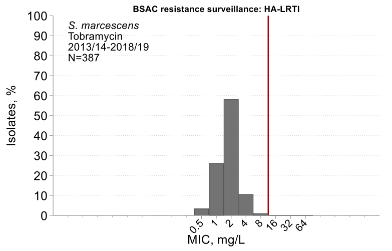

Serratia liquefaciens

Cefotaxime

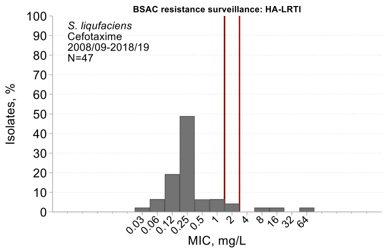

Ceftazidime

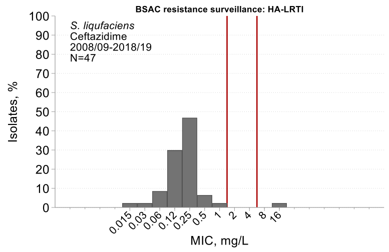

Ciprofloxacin

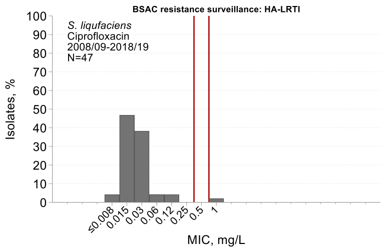

Gentamicin

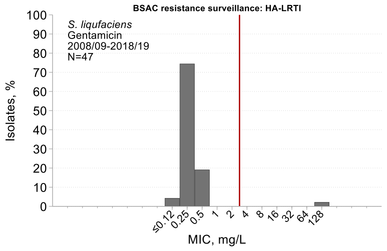

Imipenem

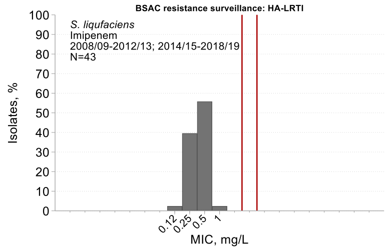

Piperacillin/tazobactam

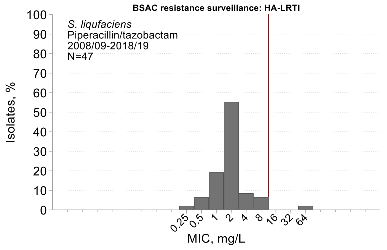

P. mirabilis

Proteus mirabilis

Amikacin

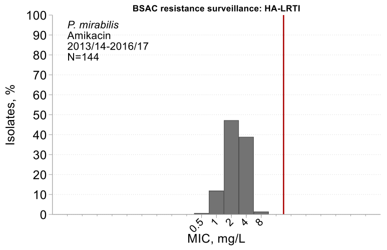

Amoxicillin

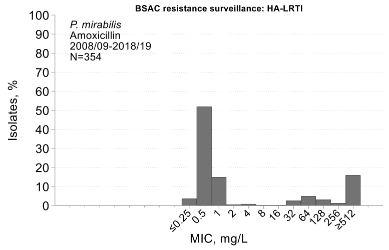

Co-amoxiclav (2:1 ratio)

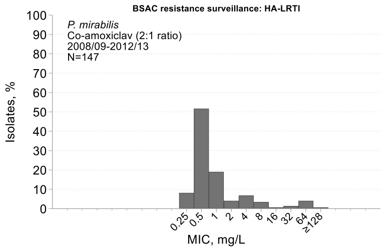

Co-amoxiclav (2 mg/L clavulanate)

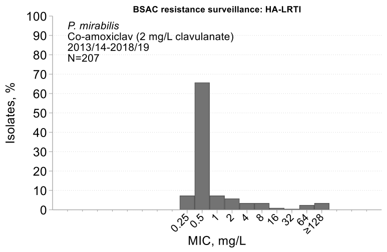

Cefotaxime

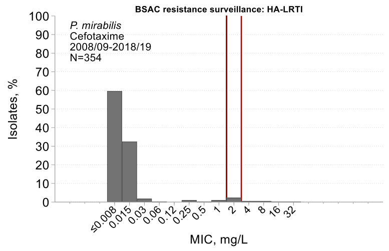

Ceftazidime

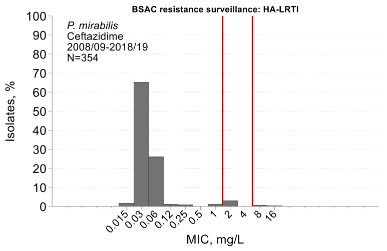

Ceftazidime/avibactam

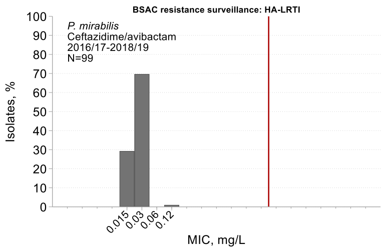

Ceftobiprole

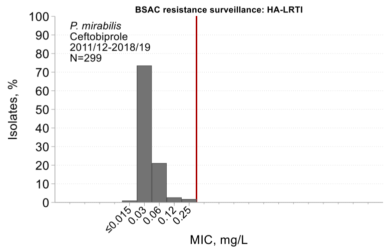

Ceftolozane/tazobactam

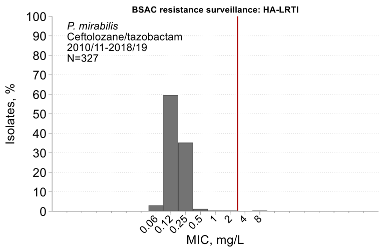

Proteus mirabilis

Cefuroxime

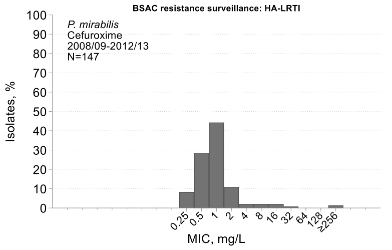

Ciprofloxacin

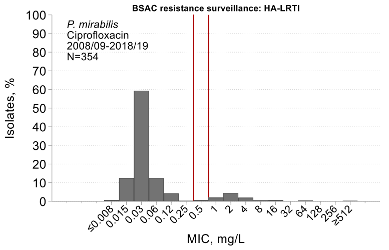

Ertapenem

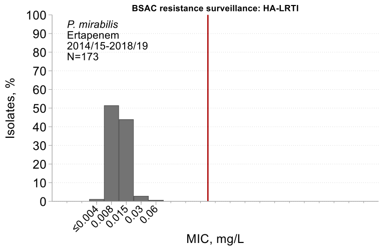

Gentamicin

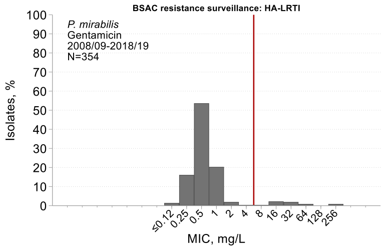

Imipenem

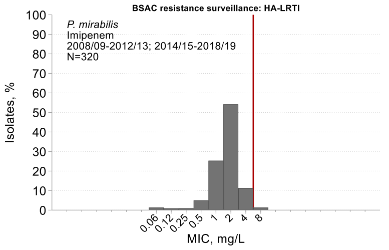

Imipenem/relebactam

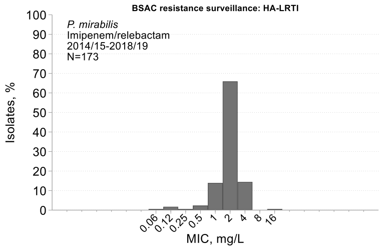

Meropenem

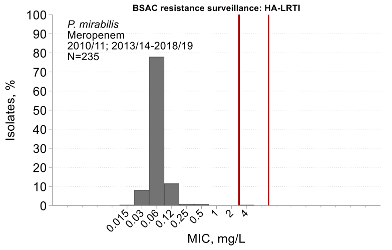

Piperacillin/tazobactam

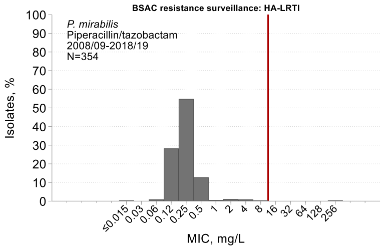

Tigecycline

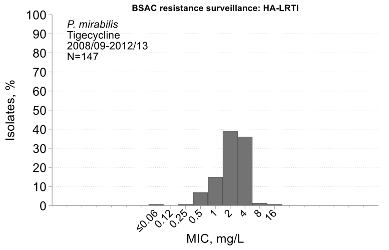

Tobramycin

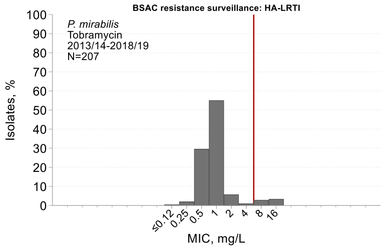

M. morganii

Note small or very small numbers of *M. morganii* (≤73)

Morganella morganii

Amikacin

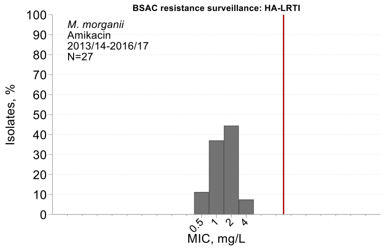

Amoxicillin

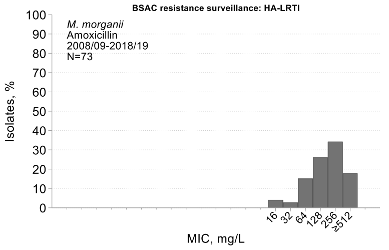

Co-amoxiclav (2:1 ratio)

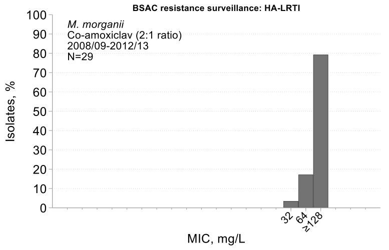

Morganella morganii

Co-amoxiclav (2 mg/L clavulanate)

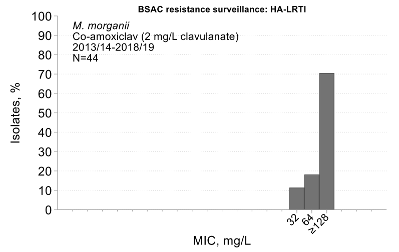

Cefotaxime

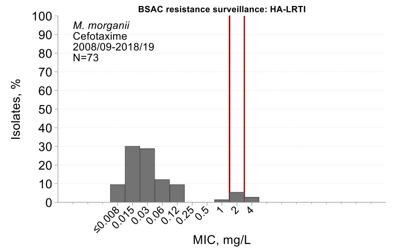

Ceftazidime

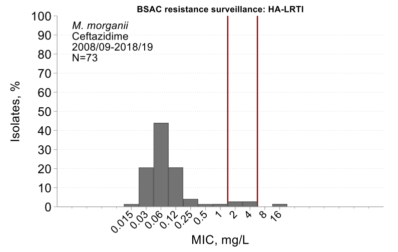

Ceftazidime/avibactam

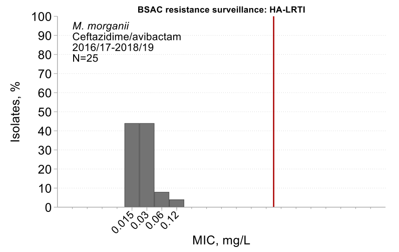

Ceftobiprole

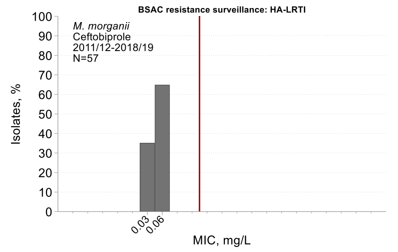

Ceftolozane/tazobactam

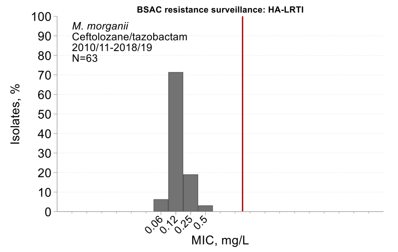

Cefuroxime

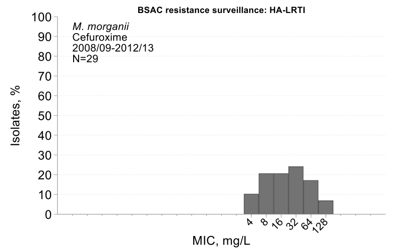

Ciprofloxacin

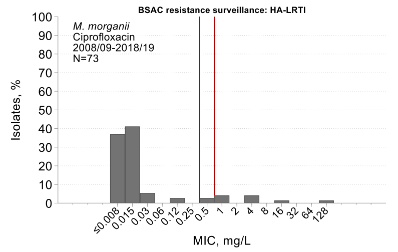

Ertapenem

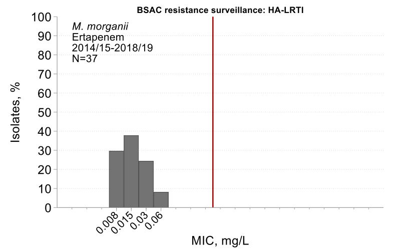

Gentamicin

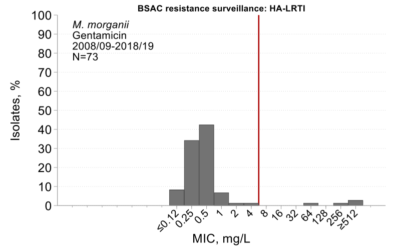

Imipenem

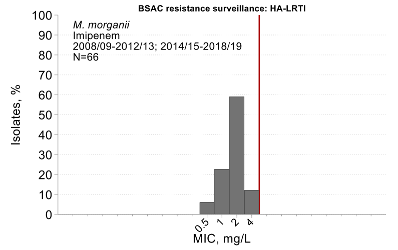

Imipenem/relebactam

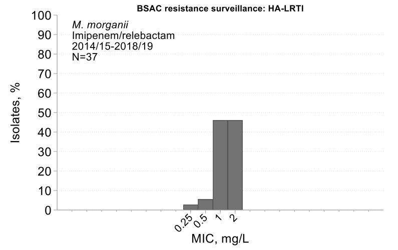

Meropenem

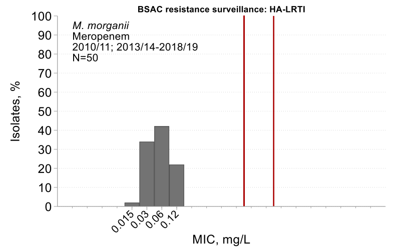

Piperacillin/tazobactam

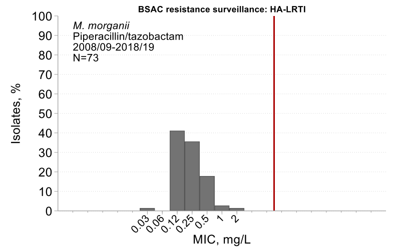

Tigecycline

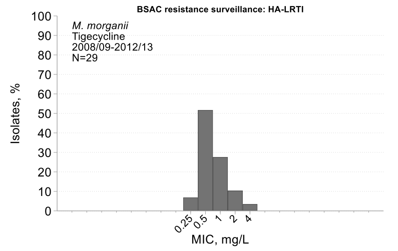

Tobramycin

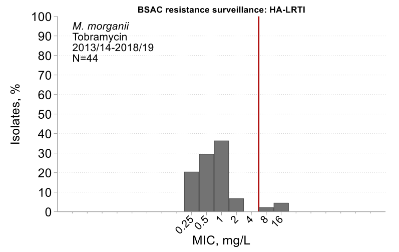

Citrobacter spp.

C. koseri	*C. freundii*

Amikacin

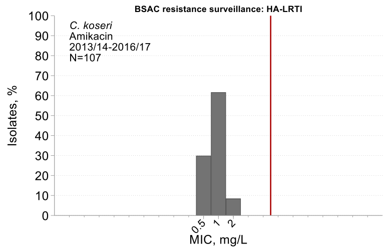

Amikacin

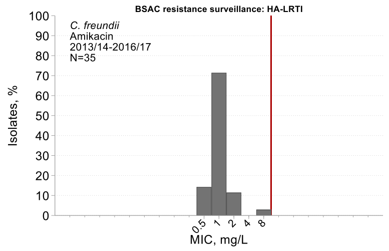

Amoxicillin

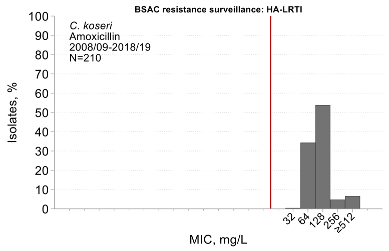

Amoxicillin

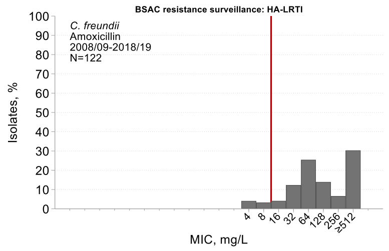

Co-amoxiclav (2:1 ratio)

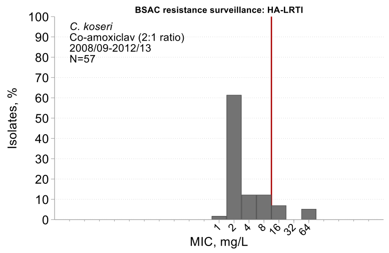

Co-amoxiclav (2:1 ratio)

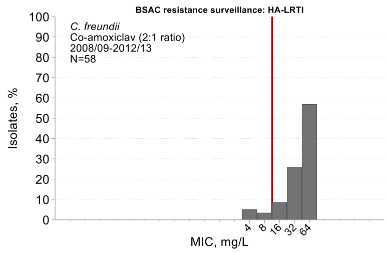

Co-amoxiclav (2 mg/L clavulanate)

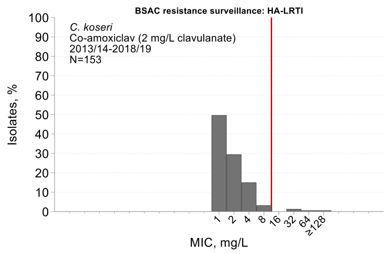

Co-amoxiclav (2 mg/L clavulanate)

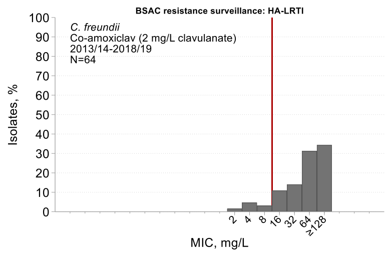

Cefotaxime

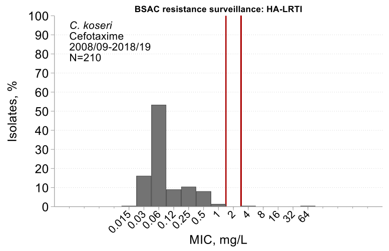

Cefotaxime

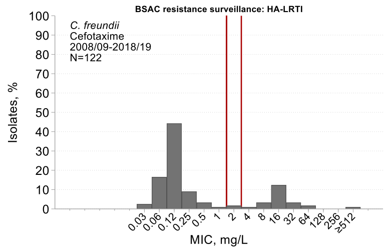

Ceftazidime

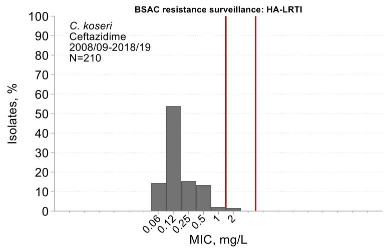

Ceftazidime

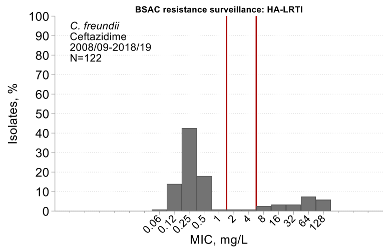

C. koseri	*C. freundii*

C. koseri: Ceftazidime/avibactam

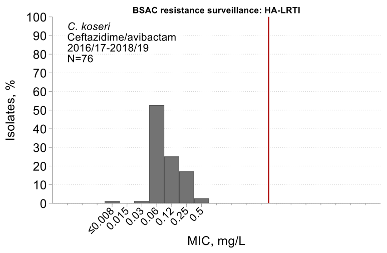

C. freundii: Ceftazidime/avibactam

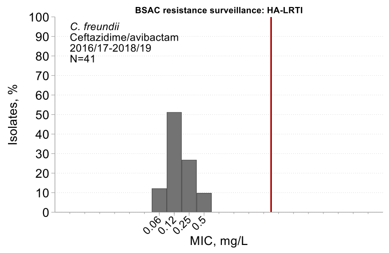

C. koseri: Ceftobiprole

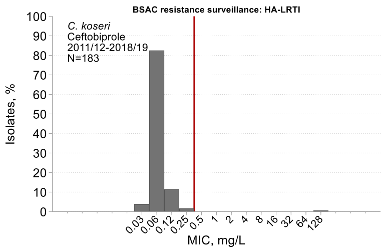

C. freundii: Ceftobiprole

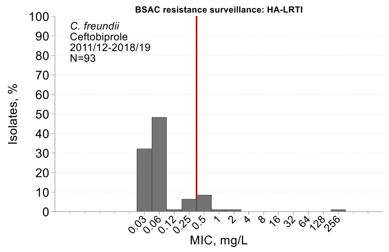

C. koseri: Ceftolozane/tazobactam

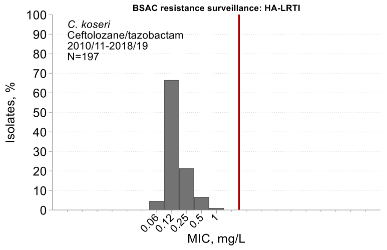

C. freundii: Ceftolozane/tazobactam

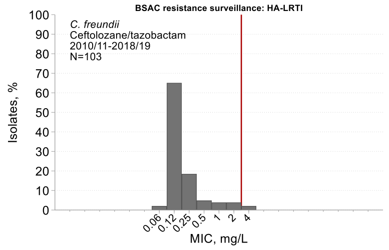

C. koseri: Cefuroxime

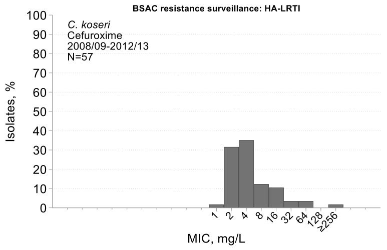

C. freundii: Cefuroxime

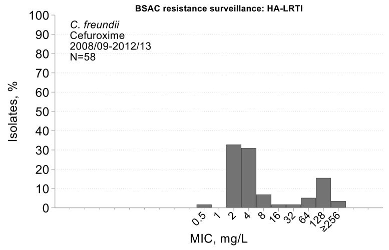

C. koseri: Ciprofloxacin

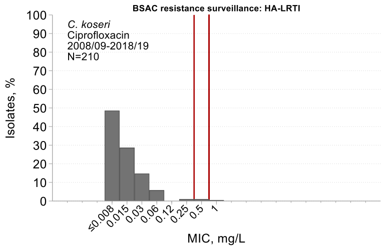

C. freundii: Ciprofloxacin

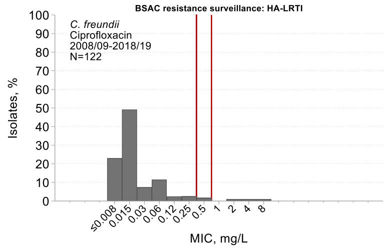

C. koseri: Colistin

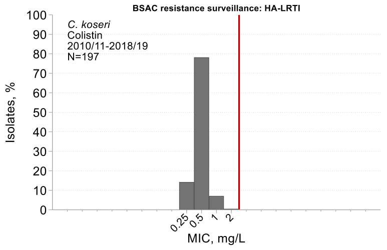

C. freundii: Colistin

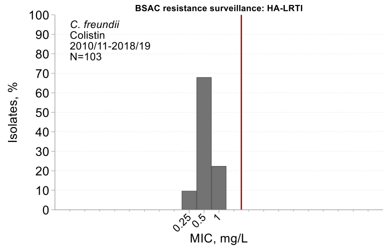

<table>
<tr><th>C. koseri</th><th>C. freundii</th></tr>
<tr><td>

Ertapenem

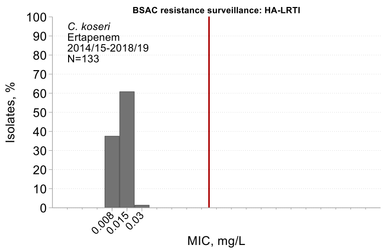

</td><td>

Ertapenem

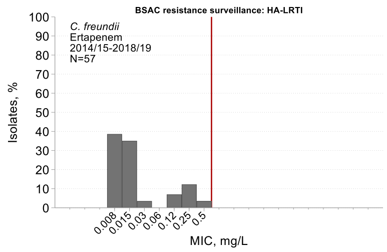

</td></tr>
<tr><td>

Gentamicin

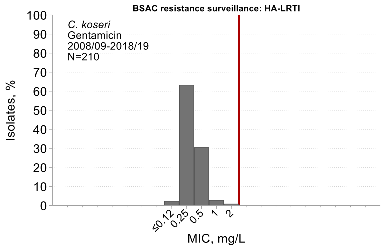

</td><td>

Gentamicin

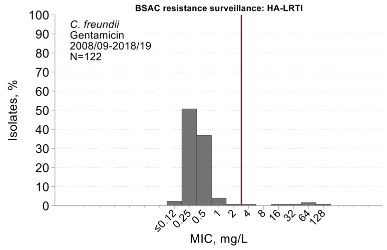

</td></tr>
<tr><td>

Imipenem

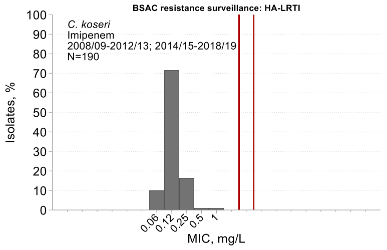

</td><td>

Imipenem

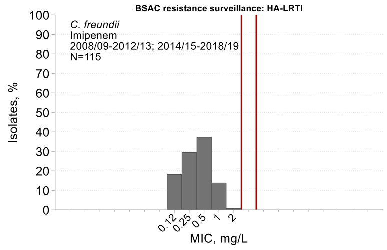

</td></tr>
<tr><td>

Imipenem/relebactam

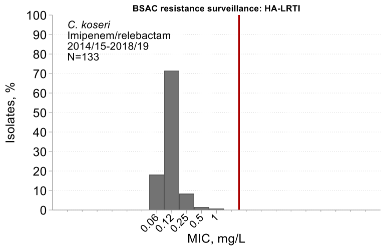

</td><td>

Imipenem/relebactam

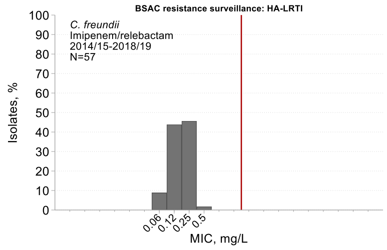

</td></tr>
<tr><td>

Meropenem

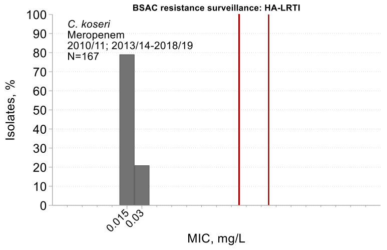

</td><td>

Meropenem

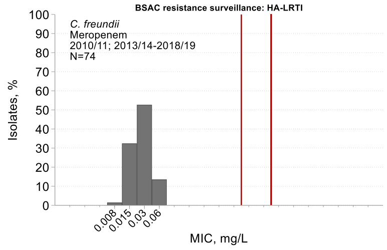

</td></tr>
<tr><td>

Piperacillin/tazobactam

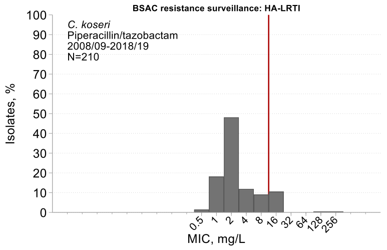

</td><td>

Piperacillin/tazobactam

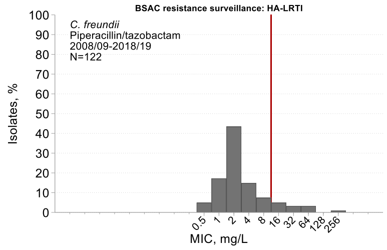

</td></tr>
</table>

<table>
<tr><th>C. koseri</th><th>C. freundii</th></tr>
<tr><td>

Tigecycline

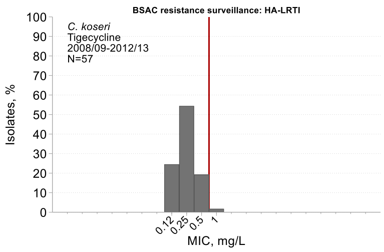

</td><td>

Tigecycline

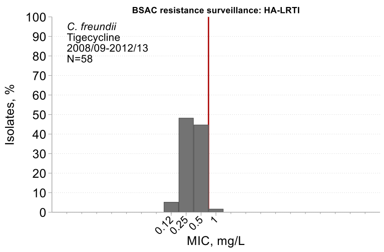

</td></tr>
<tr><td>

Tobramycin

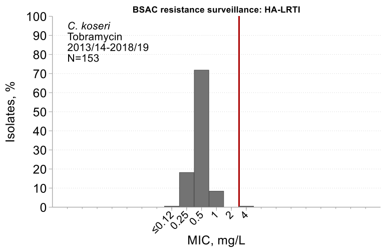

</td><td>

Tobramycin

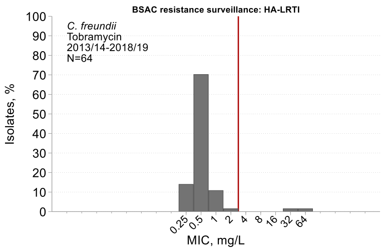

</td></tr>
</table>
